# The Potential Valorization of Corn Stalks by Alkaline Sequential Fractionation to Obtain Papermaking Fibers, Hemicelluloses, and Lignin—A Comprehensive Mass Balance Approach

**DOI:** 10.3390/polym16111542

**Published:** 2024-05-30

**Authors:** Adrian Cătălin Puițel, Georgiana Bălușescu, Cătălin Dumitrel Balan, Mircea Teodor Nechita

**Affiliations:** Faculty of Chemical Engineering and Environmental Protection “Cristofor Simionescu”, “Gheorghe Asachi” Technical University of Iasi, Bd. Prof. Dimitrie Mangeron, No. 73, 700050 Iaşi, Romania; adrian-catalin.puitel@academic.tuiasi.ro (A.C.P.); catalin-dumitrel.balan@academic.tuiasi.ro (C.D.B.)

**Keywords:** corn stalks, papermaking pulp, alkaline extraction, paper strength, antioxidant activity

## Abstract

The current study deals with an examination of strategies for the sequential treatment of corn stalks (CSs) in an integrated manner aiming to obtain papermaking fibers and to recover both lignin and hemicelluloses (HCs). Several pathways of valorization were experimentally trialed, focusing on getting information from mass balance analysis in an attempt to reveal the potential outcomes in terms of pulp yield, chemical composition, and papermaking properties such as tensile and burst strength. The raw lignin amounts and purity as well as separated hemicelluloses were also characterized. In this work, pulp yields in the range of 44–50% were obtained from CSs, while lignin and hemicelluloses yielded maximum values of 10 g/100 g of CS and 6.2 g/100 g of CS, respectively. Other findings of mass balance analysis evidenced that besides the papermaking pulp, the lignin and HCs also have interesting output values. The recovered lignin yield values were shown to be less than 50% in general, meaning that even if 67 to 90% of it is removed from CSs, only about half is recovered. The removal rates of hemicelluloses were found to be in the range of approx. 30 to 60%. About 15 to 25% of the original HCs could be recovered, and polysaccharides-based products with 67 to 75% xylan content could be obtained. Some key opinions were developed regarding how the mass balance could turn as a result of the chosen CS valorization set-up. The determined antioxidant activity showed that both lignin and hemicelluloses had interesting values for IC_50_.

## 1. Introduction

Corn (maize, *Zea mays*) has been known for millennia and nowadays is one of the most cultivated plants worldwide [1]. As a result, the problems associated with corn waste are far from being new. At a small scale, such as individual producers or micro-farms, the corn waste problem is simple to solve; however, at a large scale, where massive amounts are produced each year, things become more complicated. But given the outlook for the world’s resources in light of the energy crisis, the depletion of fossil fuels, climate change, and the continuous tightening of environmental legislation, agricultural wastes in general (not just corn waste) are slowly shifting from residues to by-products, and from liabilities to commodities [2]. The potential industrial uses of corn stalks and cobs for the production of paper, charcoal, furfural, composite fillers, and different chemicals was first mentioned by L.K. Arnold in a groundbreaking work that was published almost a century ago [3].

The valorization of corn crop residues can be divided into two categories: conventional methods, which require minimal processing to yield low-value goods, and modern methods, which require moderate to high processing levels to yield high-value products. In the “conventional approach”, there are three basic ways to deal with corn agricultural waste: silage conversion [4], soil integration before or after incineration [5], and, in some regions, as a fuel source [6]. The modern approach is to convert this abundant, renewable, and unavoidable resource into value-added products. Several reviews that highlight the benefits and opportunities of turning corn crop wastes into profitable goods are available in the literature [2,5,7,8,9,10,11,12,13,14]. The potential uses of corn waste as raw materials in different industrial domains are briefly illustrated in Table S1.

The processing degree of the corn waste is particularly important since it requires specific equipment and includes additional production costs [15]. The technical challenge for most of the modern methods is to overcome the well-known biomass recalcitrance [16,17,18] produced by the interlinking between the lignin, the cellulose, and the hemicelluloses—the main constituents of the raw material. Unlike the straws of other extensively produced crops waste (straws), which have generally consistent structure (wheat, rice), the aerial section of the maize plant is more complex, with considerable structural and compositional differences among the various parts (e.g., stalk, leaves, cobs, husks, and silk) [19,20]. Moreover, corn waste can be produced in two ways: first, while harvesting (stalks, leaves, husks, and cobs), and second, during processing (cobs and sometimes husks) [10]. Over time, valorization processes have been identified for the entire plant as well as for the individual components (see Table S1).

The multitude of potential applications for value-added products and the heterogeneity of corn plant constituents [21], each with a characteristic recalcitrance [22], are among the factors that have contributed to the remarkable development of processing methods. Another technical aspect to consider before designing a technological process for corn waste valorization is that the chemical composition of the raw material is variable, depending on the plant variety, weather conditions, fertilization and other chemical treatments, harvesting period, storage period and conditions, and other factors [23]. That explains the abundance of technological approaches reported in the literature (see Table 1), where various pretreatment methods (designed to reduce the corn residue recalcitrance) are associated with different extraction techniques to increase yields. Optimization methodologies are often employed to find the ideal processing parameters [24,25,26,27].

One of the challenges that must be overcome in real-world applications based on CS wastes in terms of process performance and equipment design is the complexity and inconsistency of raw material chemical composition. Understanding the material inputs, outputs, and accumulations given by the mass balance is the first step in designing and optimizing processes and equipment.

This study aimed to determine the best-suited strategy for the complete valorization of CSs from the perspective of obtained papermaking pulp as well as the quality of the obtained co-products lignin and hemicelluloses. Five CS treatment configurations were proposed and evaluated to get optimal results in terms of pulp yield, amounts, and the purity of recovered lignin and hemicelluloses. To simplify the procedure, and to alleviate the need for inter-stage washing, all the proposed scenarios were based on using sodium hydroxide as a single chemical agent. Special attention was paid to the liquid streams’ chemical composition and overall mass balance. To the authors’ knowledge, such an experimental strategy has never been attempted and reported.

In brief, our work focused on the analysis of various configurations of CS treatment, which included hot water treatment (HWT), mild alkaline extraction (AE), and finally, soda pulping (SP). This investigation addressed issues such as the composition of the resulting solid materials and the treatment of liquid streams resulting from the CS experimental processing, and also from lignin and hemicellulose separation stages. Most of the treatment stages involved the usage of alkali, in particular, sodium hydroxide, as both an extractive and delignification agent. We also aimed to reveal both the potential advantages and drawbacks of hot water treatment and alkaline extraction as preliminary pulping treatments. The combination of alkaline pretreatments eliminates the inter-stage washing, therefore reducing the potential water consumption if eventually applied industrially. As previously emphasized by other authors [35,36], the washing is necessary for the removal of contamination risk in the subsequent phase, either performed by organic solvent action or at a different pH. However, in our study, washing was not eliminated as it was a necessary step after the HWT stage.

Apart from previous work that focused on finding the optimum equilibrium between the amount of extracted HCs and paper quality, this study took into consideration the analysis of both solid as well as liquid streams [37]. The obtained products were characterized from a common perspective (chemical composition) as well as from the perspective of their usage potential (papermaking properties, degree of polymerization, and antioxidant activity). The analytical results were thereafter used to achieve a mass balance which, when combined with the other mentioned findings, would reveal aspects concerning the bigger picture and support possible integration of agri-waste as biorefinery feedstocks.

## 2. Materials and Methods

### 2.1. Materials

Corn stalks consisting of a mixture of hybrid stems were provided free of charge by Romanian farmers from the harvest of 2023. After harvesting, the CSs remained for drying at room temperature to about 10% humidity. The stalks were cut into 20–50 mm pieces for hemicellulose (HC) extraction and/or pulping studies. The chemical composition determinations were performed after the CSs were ground and sieved to 0.2–0.5 mm particles.

All chemicals and reagents used were of analytical purity. Solutions of 99% purity of cellobiose, glucose, xylose, galactose, and arabinose, provided by Biochem Chemopharma (Cosne-Cours-sur-Loire, France), were used to obtain the HPLC calibration curves in the concentration range of 0.05–2 g/L.

### 2.2. Equipment

#### 2.2.1. CS Treatment, Pulp Production, Sheet Formation, and Testing

Hot water treatment (HWT), hot alkali extraction (AE), and soda pulping (SP) experiments were conducted in a 10 L stainless steel laboratory rotating digester. The used digester was electrically heated and equipped with a temperature controller.

Each experimentally obtained solid fraction (treated CSs and pulps) was washed several times and hand-squeezed to a moisture content of about 75%. They were further kept for analysis and papermaking for several days at 4 °C to avoid degradation.

Before papermaking on a Rapid Koethen laboratory sheet former ISO 5269/2 [38], the pulps were all refined at a constant number of revolutions (1500) in a Jokro mill, ISO 5264-3:1979 [39]. The beating resulted in pulps of 45–48 °SR drainage resistance, a value at which the mechanical strength properties are fully developed [37]. The obtained paper sheets were the subject of testing—in this respect, tensile strength and burst strength were determined according to ISO 1924-2:2008 [40] and ISO 2758:2014 [37,41]. The values of SCTI and CMTI were obtained after performing SCT [42] and CMT [43] tests and reporting the obtained values (kN/m and daN) for the basis weights of the tested paper.

#### 2.2.2. Analysis of Sugars

A Shimadzu Nexera LC 40D liquid chromatography system equipped with a Shodex SP0810 column (300 × 8 mm, particle size—8 µm, Resonac, Shūnan, Japan) heated at 65 °C was employed to perform the required HPLC analyses. The refractive index detector (Shimadzu RID 20A, Kyoto Japan) was set at 40 °C. The flow rate of the mobile phase (ultrapure water) was 0.6 mL/minute. The injection volumes were set between 80 to 100 µL, depending on the sample concentrations. Samples, as well as standard solution (containing cellobiose, glucose, xylose, galactose, and arabinose of analytical grade), were filtered before injection using 0.2 μm syringe PTFE filters.

#### 2.2.3. Spectroscopy

The FTIR spectra of hemicelluloses as well as lignin samples were recorded by an Agilent Cary 630 (Santa Clara, California, USA) using the potassium bromide pellets technique on disks containing finely ground samples at 1% content.

The Jasco V530 UV-VIS spectrometer (Tokyo, Japan) was used for measuring the absorbance of hydrolysate solutions at 320 nm wavelength. The values were necessary to compute insoluble acid-insoluble lignin contents. The same equipment was employed in the antioxidant activity determinations for measuring absorbance values at 517 nm.

#### 2.2.4. Other Equipment

A Sorvall GLC2 centrifuge (DuPont Instruments, Sorvall Operations, Newtown, CT, USA) equipped with an HL-4 rotor, CF value—2012, 3000 r.p.m., was used for precipitate separation (lignin and HCs). The pH and conductivity values of the liquids generated in different experimental stages were tested with a Consort 532 pH meter and a WTW 303i conductivity measurement device. The moisture content was determined with a Radwag MA 210.R Moisture Analyzer (Radom, Poland).

### 2.3. Experimental

The experimental set-up used for the CS biomass processing involved stages of hot water treatment (HWT), alkaline extraction, and finally, pulping by soda process. All experiments and determinations were performed in triplicate unless otherwise stated by the mentioned standard methods. The accepted maximum relative standard deviation value was less than 5%.

The materials recovered from each experimental method (HWT, HAE, or SP), whether solid or liquid (hot water treatment liquid, alkali extraction liquor, black liquor), were analyzed for HC, cellulose, and lignin content.

### 2.4. Corn Stalk Treatment Strategy

#### 2.4.1. HWT Treatment

Typical HWT experiments used 300 g of oven-dried (o.d.) biomass. CSs were treated in the laboratory reactor at a solid-to-liquid ratio of 1:10 for 60 min at 100 °C. The heating time was 20 min. At the end of the HWT process, the resulting liquid phase (HWT liquor) was saved for further analytical characterization. The HWT-treated corn stalks were washed, dried to a suitable moisture content (8–10%), analyzed for chemical composition, and then used for alkali extraction and soda pulping. The material loss was determined gravimetrically.

#### 2.4.2. Alkali Extraction of Corn Stalks and Soda Pulping

The AE of CSs was employed as a method for the extraction of a portion of lignin and hemicelluloses. Alkali extraction of CSs (AE) was conducted either as a single CS treatment stage (AE-denoted experiments) or as preliminary soda pulping of CSs (AE SP) or HWT-treated CSs (HWT AE SP).

The AE as a single treatment consisted of extracting polymeric forms of hemicelluloses from corn stalks with solutions containing 1% sodium hydroxide (NaOH). A total of 200 g (o.d.) CS was immersed in the NaOH solutions at a solid-to-liquid ratio of 20. The reactor was closed and heated to 100 °C and maintained at this temperature for 33 min. After the extraction, both the liquid phase, further denoted as alkaline extraction liquor (AE), and the remaining solid material were separated, washed, screened, characterized, and further processed to obtain paper sheets.

In the AE SP configuration, the AE was performed as before mentioned, but at the end of the AE duration, a volume of 1.5 L of AE liquor was removed. The experiment was continued by restarting the heating and the temperature was raised to 135 °C (heating took 10 min). This temperature was maintained for 30 min and the experiment was referred to as soda pulping (SP). At the end of the SP time, the black liquor (BL) was saved for further analysis (1.5 L). A subsequent reactor cooling step (60 °C) was employed, after which the reactor was opened to evacuate pulp (10% consistency) that was then washed and screened, characterized, and further processed to obtain paper sheets. The HWT AE SP configuration involved an identical path but used HWT-treated CSs.

Soda pulping (SP) as a single processing option of CSs was carried out in the same reactor under the following conditions: SP1 at a solid-to-liquid ratio of 1:10 and SP2 at a solid-to-liquid ratio of 1:20. In both of the SPs, the heating time was 20 min, while cooking took 30 min at 135 °C and a constant pressure of 0.2 MPa. The corresponding white liquor had an initial 1% NaOH concentration.

In all the experimental set-ups, the obtained pulps were wash-screened (2 × 0.5 mm screen gap) and used for further analysis and sheet formation for strength testing. The total solid yield was determined by gravimetric means and calculated using Equation (1), while the screening rejects were dried and weighed to determine the relative amount of rejects R (%)—Equation (2). The difference between SY and R leads to the value of the screened yield.

(1) $$SY\left(\%\right)=\frac{m_{f}}{m_{i}}\cdot 100,$$

(2) $$R\left(\%\right)=\frac{m_{r}}{m_{i}}\cdot 100,$$

where SY (%) represents the solid yield; m_i_ is the o.d. weight of the initial CS biomass; m_f._ is the o.d. weight of the CSs after the treatment or treatment sequence; and m_r_ is the amount of rejects that resulted from screening.

The severity factor values (SF) corresponding to each CS processing set-up were mathematically established by using Equation (3). The SF allows the combination of the temperature and duration of treatment into a single parameter and may be used to achieve comparative purposes [44,45].

(3) $$SF=\log_{10}\left(\tau *e^{\frac{T-100}{14.75}}\right),$$

where τ is the process time at selected temperature T.

#### 2.4.3. Separation and Purification of Lignin and HCs from Alkaline Process Liquors

The ethanol precipitation method was used to separate hemicellulose from the liquors obtained during the experimental procedures. In brief, 200 mL liquor (alkaline extraction) samples were neutralized to pH 4.5 with acetic acid. A first centrifugation stage was performed to remove the precipitated lignin. The raw lignin (RL) was removed from the centrifuge vials and vacuum oven-dried at 55–60 °C to account for separation yields. The resulting supernatant volume was reduced to 50 mL by using vacuum distillation and subsequently mixed with 2 volumes of analytic purity ethanol (96%) and stored at −18 °C for 60 min. The precipitated HC samples were separated by centrifugation at 3000 r.p.m. for 5 min. Next, two rounds of ethanol washing were performed. Following each washing, a 5 min centrifugation was carried out at 3000 r.p.m. to separate the solid from the ethanol. The crude HC samples were dried at 50 °C before further analysis. The liquid streams that resulted after hemicellulose separation were saved and subjected to ethanol recovery by vacuum distillation using a rotary unit and further referred to as ethanol recovery liquid streams (ERLSs).

### 2.5. Characterization Methods

#### 2.5.1. CS, Treated CsS, and Pulp Characterization

Several analytical procedures were used to determine the chemical composition of raw CSs in terms of both major components (polysaccharides and lignin) and minor components: ash—TAPPI T 211 om-02, 200; hot water extractives—TAPPI T 207 om-88 [46]; organic solvent extractives T 204 cm-97 [47]; and acetone extractives (AEs)—TAPPI T280 pm-99 standard (2000) [48]. While acid-insoluble lignin (AIL) and acid-soluble lignin (ASL) were determined using the sulfuric acid two-stage hydrolysis method specified by the NREL/TP-510-42618 method [49], the major polysaccharide components (cellulose and hemicellulose) of the biomass and the obtained papermaking fiber were determined following an adaptation of the procedure described by Sluiter et al. [50]. The adaptation involved neutralizing the hydrolysate from a G3 crucible filter to pH 5.6 before HPLC analysis. The conversion of monomer concentrations to their corresponding polymer concentrations was realized by considering the ratio between the molecular weights of the anhydro-sugar unit and the sugar unit (162/180 = 0.9 for C6 sugars and 132/150 for C5 sugars).

Calculations based on Equation (4) were used to account for the removal rates of CS components during treatment.

(4) $$Removalrate\left(\%\right)=\frac{m_{i}*X_{i}-m_{f}*X_{f}}{m_{i}*X_{i}}\cdot 100,$$

where m_i_ is the mass of initial raw CSs subjected to treatment, X_i_ is the initial percentage of the component (glucan, xylan, galactan, arabinan, and lignin), m_f_ is the final mass of either HWT-treated CSs or pulp subjected to treatment, and X_f_ is the final percentage of the components (glucan, xylan, galactan, arabinan, and lignin).

Determination of silicon content in the ash was performed by using the method provided by the literature [51,52]. In brief, the method consists of treating the 10% nitric acid dissolved ash (resulting from previous sample combustion) with an acidic solution of ammonium heptamolybdate ((NH_4_)_6_Mo_7_O_24_) solution to generate silicomolybdic acid (H_4_SiMo_12_O_40_). This is further reacted with ascorbic acid to achieve the reduction of silicomolybdic acid and the formation of a blue-colored complex. The final step is recording the absorbance of the solution at 600 nm. A calibration curve was constructed by using appropriate dilutions of 1000 ppm silicon standard solution (Supelco (Bellefonte, PA, USA)).

The average degree of polymerization of the obtained hemicellulose samples was determined by using viscosity data as described in the literature [53]. In brief, the samples were dissolved in a 0.04 M cupriethylenediamine (CED) solution, and the intrinsic viscosity was determined at 25 °C. The Staudinger−Mark–Houwink method (xylan in CED) is based on Equation (5) [53,54,55].

(5) $$\left[\eta \right]=2.2\cdot 10^{-2}\cdot {DP}^{0.72},$$

The obtained pulps’ DP was established after determining the intrinsic viscosity (Equation (6)) in 0.5 M CED solution [56].

(6) $$\left[\eta \right]=2.28\cdot {DP}^{0.76},$$

Following washing and refining (beating), the obtained pulps were transformed into paper sheets that were subjected to analysis of tensile strength (ISO 1924:2008) [40] and burst strength (ISO 2758:2004) [41].

#### 2.5.2. Characterization of Liquor Samples, Raw Lignin, and Crude Hemicellulose Samples

The extracted liquor samples from the CS treatment stages were initially cooled and filtered to remove any solids (fibers or debris). Before using them in the separation of lignin, they were subjected to measurements of pH and conductivity and for the determination of total solids content, ash, organic material content, lignin, and sugars. The solids content was determined gravimetrically after evaporation at 105 °C on an evaporation dish. The remaining solid materials were combusted at 550° to determine minerals while the total organic material content was computed by difference.

The carbohydrates present in liquor samples were analyzed after they were treated with 4% sulfuric acid (60 min at 121 °C) according to NREL (LAP) TP-510-42623 [57]. Samples of 10 mL of liquor were mixed with 72% sulfuric acid and further diluted with distilled water. Afterwards, the treatment was continued by complete hydrolysis which was achieved by incubation at 121 °C for 60 min. Following hydrolysis, the samples were cooled and filtered on a G3 crucible to determine the amount of acid-insoluble lignin. The filtrate was collected and tapped to 100 cm^3^ in a volumetric flask and absorbance at 320 nm was recorded to determine the amount of acid-soluble lignin. Portions of the same filtrate were neutralized and HPLC was analyzed for the concentration of monomeric sugars.

The phenol index for ERLSs was determined according to ISO 6439:1990 [58] which involves distillation and subsequent reaction of distilled free phenols with 4-aminoantipyrine. The absorbance value of the generated colored complex is measured at 510 nm.

The COD in washing water was determined by using the ISO 15705:2002 [59] specifications. However, the results were computed by taking into account the volumes of washing water and further obtaining the values of total COD generated in each experimental configuration.

#### 2.5.3. Evaluation of the Antioxidant Activity

Evaluation of antioxidant activity was performed by an adapted version of DPPH (2,2′-diphenyl-1-picrylhydrazyl radical) assay [60,61]. The adaptation consisted of the following: the DPPH solution was initially prepared by using 96% ethanol at a concentration of 0.2 mM; the samples of purified lignin and isolated HC samples were firstly dissolved in distilled water to make up the stock solutions; afterward, dilutions were performed in a volumetric flask to end up in concentration ranges of 0.5–3.5 g/L for HCs and 0.5–1 g/L for purified lignin samples. A method blank was prepared by diluting DPPH 0.2 mM to 0.1 mM and its absorbance was recorded as *A*_0_. Samples’ absorbance measurements (*A_i_*) were recorded after mixing 3 mL of sample solution with 3 mL of 0.2 mM DPPH and this being placed in a dark thermostatic oven for 30 min at 37 °C. The blank was treated similarly. The inhibition, I(%), was calculated as indicated by Equation (7).

(7) $$I\left(\%\right)=\frac{A_{0}-A_{i}}{A_{0}}\cdot 100$$

where: A_0_ is the absorbance at 517 nm that was recorded for the blank; A_i_ is the absorbance of samples at the same wavelength. Subsequently, recording of the absorbance values at different concentrations gave the dependence of I (%) vs. C (g/L or mg/L) that was plotted to reveal the IC_50_ (g/L or mg/L).

## 3. Results and Discussion

### 3.1. Chemical Composition of CSs and Treated CSs, and the Mechanical Strength of Pulps

**Figure 1:**
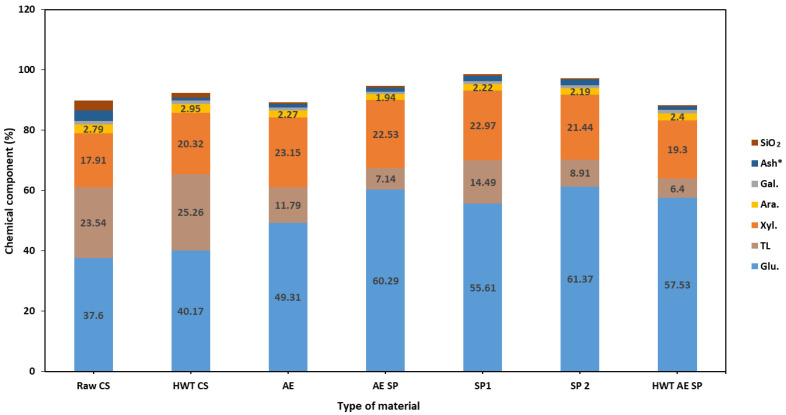
Chemical composition (% wt.) of corn stalks HWT-treated and solid material—papermaking fibers (pulp). The Ash* stands for ash content value (%) corrected by subtraction of silicon dioxide SiO_2_.

The chemical composition of the CSs showed different extents of changes in all the experimental situations. Figure 1 reveals the evolution of the contents of the chemical components of CSs as well as of the various solid fractions and cellulosic fibers resulting from the performed treatments. The main components of initial CSs are cellulose, hemicellulose, and lignin, and the obtained values were comparable with other literature data [62,63]. As compared to our previous work [37], the cellulose content showed lower values for cellulose and xylan contents but higher for lignin. Several elements leading to the differences in chemical composition might be corn variety, precipitation/irrigation level, fertilization, and harvesting period [64,65,66].

A relatively mild treatment such as HWT affects the chemical composition of the CSs—an increase in cellulose, xylan, and acid-soluble lignin contents may be easily noticed, while the content of ash and acid-soluble lignin is decreased. Although existing studies report that HWT is associated with selectivity for xylan extraction and glucan stability under such treatment [67], the main reason for such results in our study was the dissolution of water-soluble components during the treatment, as revealed by the low concentration values in the HWT liquor. Acid hydrolysis also occurs at such temperatures, mainly resulting in HC loss and cellulose depolymerization, but not in significant xylose release, as also indicated in Table 2. According to Kang et al. [68], the degradation of xylan, and the release of xylose and its further degradation to furfural and formic acid, occur at higher temperatures (and corresponding higher severity of treatment).

**Figure 2:**
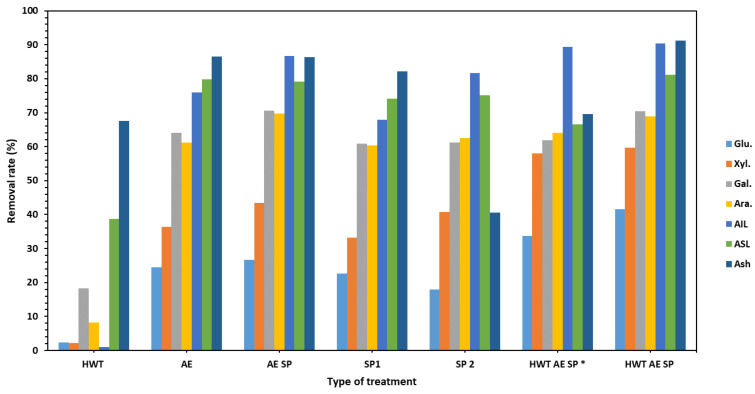
The corresponding removal rate (%) of components from CSs as a result of the performed treatments. The HWT AE SP * values of the removal rates relative to after HWT treated biomass components content while HWT AE SP represent values of the removal rates relative to the initial raw untreated CS biomass.

Performing the AE treatment on the CSs led to much different results: a higher increase in polysaccharides content was noted while the AIL significantly decreased. When comparing the extent of lignin removal vs. polysaccharides loss, the conclusion that the performed AE treatments are mainly selective in lignin removal may be drawn—the lignin removal rates ranged from 67% to about 90% in the case of HWT AE SP. Further continuation of the process by subsequent soda pulping (SP) leads to further reduction in the lignin content of the obtained papermaking pulp. Similar aspects may be noticed for the rest of the studied treatment strategies. In the case of SP1, although the sodium hydroxide concentration had the same value of 1%, the liquid-to-solid ratio was 10. The chemical composition of the resulting fibrous material was obviously different from the situation of SP2 where the liquid-to-solid ratio was 20. This is a result of the fact that in SP1 the alkali charge was only 10% while in SP2 the value of the active alkali charge was 20%. In fact, the lignin removal value (~68%) in the case of SP1 remained even lower than in the AE and AE SP (75–67%) treatments, confirming the importance of alkali in the delignification process. The loss values for other components of the CSs may be observed in Figure 2.

Analyzing the values presented in Table 2, several aspects may be observed regarding the importance of the CS treatment conditions in terms of severity. The hemicellulose RY values are indeed influenced by the SF, although the variation is not quite well correlated. In this particular experiment, the SF value has a clear influence on the pulp yield value. The overall conclusion concerning the removal of HCs is that a large portion of them was retained in the CS pulp with further beneficial effects on strength properties. The total pulp yield values were acceptable in most of the studied configurations, but the sorted yield showed promising results only for the AE SP and HWT AE SP. However, as revealed by Figures S1–S5 in the Supplementary Materials, all pulps could be used for papermaking but a refining stage was necessary.

One other important positive aspect of the introduction of the actual pulping preliminary treatments of CSs seems to be the reduction in ash content which also correlated with silicon content decrease. The ash and silicon contents are some of the most important problems associated with non-woods in general and with agri-waste in particular when used as a feedstock for papermaking pulp production. Due to their precipitation, they are the main impediments in the evaporative chemical recovery process and further black liquor burning in a recovery burner [22,51,69]. In our study, the silicon content (expressed as SiO_2_) of the analyzed samples showed values of approx. 3% in raw CSs down to 0.4% in the HWT AE SP sample. The HWT and AE alone reduced the silicon content of the CSs to 1.42 and 0.55%, respectively.

Table 2 contains data on the HC degree of polymerization (DP) that correlate relatively well with the increasing values of the SF. Furthermore, Table 3 reveals the viscosity average degree of polymerization of pulps, the mechanical strength properties together with total yield, and the rejects obtained for each of the CS processing experimental scenarios. The purity and recovery yield of HCs (calculated as the ratio between removed HCs and actual recovered HCs) seemed to be less dependent on the SF. In the case of pulps, the viscosity average DP seemed to be less dependent on the SF, except for the HWT AE SP sample, for which it proved to be significantly lower than for the other samples. The obtained values were lower than those reported for pulps obtained from softwood [70].

Tensile index (TI) and burst index (BI) obtained values (Table 3) were found to correlate well with the polysaccharides content and with the degree of delignification. Studies in the literature indicate that the presence of lignin may hinder the formation of hydrogen inter-fiber bonds which lead to reduced mechanical paper strength [71]. The mechanical strength properties showed considerably better values when compared to values obtained for a sample of recycled industrial paper (fluting, 120 g/m^2^) (TI of 24.3 Nm/g and BI of 1.72 kPam^2^/g), proving the potential of the obtained papermaking CS fibers as either raw materials for packaging paper or as reinforcements, as already proposed by Hagel and Schütt [72]. Photos of sample laboratory paper sheets are presented in the Supplementary Materials, Figures S1–S5. The values for SCTI varied in a narrow range and mostly indicated that these samples are more suitable for corrugated board paper production when compared to those obtained for recovered-paper-produced fluting (15 N·m/kg). The values for CMT were shown to be similar or about 10–15% higher than those of that same fluting (1.3 N·m/g).

On the other hand, the SF seems to have much more impact on the yield values. By analyzing the pulp yield, it may be observed that the most favorable situation is represented by the combination of AE and subsequent SP which yielded 45% solid material with a corresponding rejects value of 4.5%. In this scenario, the strength properties showed a maximum value. Using the HWT treatment led to a lower yield value but also a drop in rejects to about 3.45%.

### 3.2. Chemical Composition of Liquid Streams and Co-Products

**Figure 3:**
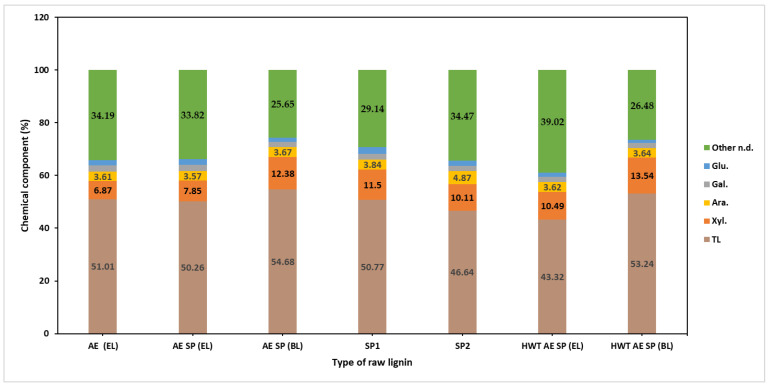
Chemical composition of the recovered raw lignin samples.

**Figure 4:**
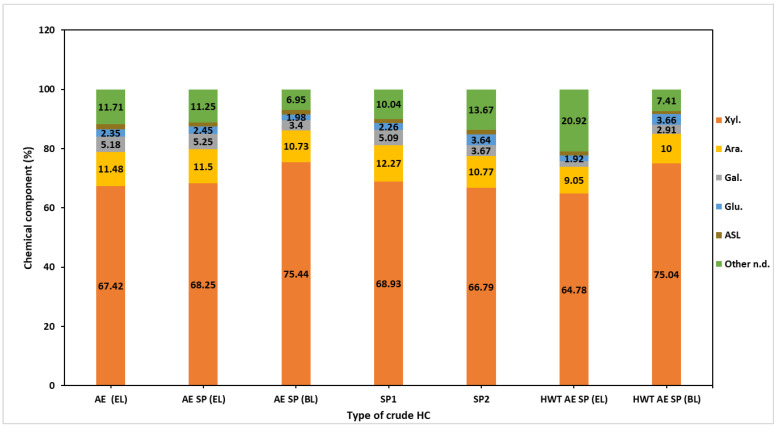
Chemical composition of the crude hemicelluloses isolated from experimental process liquors.

Table 4 reveals the chemical composition of the obtained process liquors, while Figure 3 and Figure 4 refer to the chemical composition of the recovered raw lignin and crude hemicellulose samples. Except for the HWT liquor, Table 4 shows relatively similar values for the end pH of studied liquid samples, aside from the BL obtained from the SP1 experimental situation. The relatively low acidic pH value of HWT liquor is a consequence of the formation of residual acids formation [73]. Although not completely linked to NaOH concentration, an examination of the conductivity values showed that a considerable conductivity drop occurs during the AE and also the pulping stage. The conductivity values might be a rough indicator of soda consumption as a result of chemical reactions taking place during the process [74].

It may be observed that the AE SP (BL) recovered lignin sample had the highest lignin content but also the highest polysaccharide content. A similar value was obtained for the HWT AE SP (BL) lignin which also showed high xylan content. The lignin samples obtained from the SP1 and SP2 experiments showed considerable differences in purity and the results proved to be somehow contradictory—the only explanation possible for the lower purity lignin of the SP2 sample is the extent of degradation under the conditions of a higher alkali charge [75].

When switching from AE to AE SP, it may be observed that both the lignin and also the dissolved polysaccharides concentration almost double their values. This is both a result of volume reduction, but also from the additional lignin and PS release from the vegetal tissue. In fact, the AE stage, although having some delignification effect at low temperature, combines the roles of lignin and hemicellulose extraction but also the role of impregnation. In general, lignin represents about 32–45% of the dissolved organic solids while polysaccharide concentration values indicate similar percentages.

In the extraction of HWT-treated CSs, although the values of the organic matter concentration seemed lower than in the other process set-ups, the share of lignin and total polysaccharides showed higher values of up to 43–44%. Several other aspects may be noticed regarding the chemical components’ concentrations. In the case of the HWT samples, the concentrations of lignin and xylan proved to be lower than in the AE SP resulting liquors. Further observation shows that the OMC also follows a similar trend. The SP1 liquor seems to present higher values for all the components, but readers are advised to notice that the values were obtained for a lower liquid-to-solid ratio. In reality, the amounts of the chemical components in black liquor are lower as SP1 was performed at a lower alkali charge.

The chemical composition of the ethanol recovery liquid streams (ERLSs) was taken into consideration to reveal the amounts of chemical components in the CSs that might exist as a result of the separation operations. Their values are shown in Table 5. It may be observed that significant amounts of both sugars and lignin co-exist. In fact, the compositional data show that more than half of an ERLS consists of both lignin and polysaccharide degradation chemicals.

The ERLSs recovered from the processing of black liquor showed a tendency toward higher values in terms of AIL concentration, a fact that correlates with the amount of removed lignin. The concentration of the total polysaccharides, mainly consisting of oligomers of anhydro-xylose units, which did not precipitate in ethanol, increased with the increase in the severity of the treatment, probably as a result of the degradation extent. In fact, the increase in the severity of the treatment also negatively affects the HC recovery yields, as previously noted [37]. Analysis results for the chemical composition of the recovered lignin samples can be seen in Figure 3. In general, the purity value for the alkaline CS lignin specimens was found to not exceed 55%, which correlates with other reported data such as those of Manorma et al. [76]. This was a result of the chosen separation procedure that involved pH reduction to a value of 4.5. It is relatively well known that the pH value has a considerable effect on both the yield and purity of the recovered black liquor lignin [77,78]. In brief, lowering the pH of lignin separation leads to better yields in lignin separation but reduces the purity of the raw lignin due to precipitation of HCs [79].

The chemical composition of the raw hemicellulose sample (Figure 4) showed a content of xylan ranging from 67% to 75%. In comparison to our previously published results, the purity of the HC samples in the current investigation was enhanced [37] as a direct result of the modification of the separation procedure consisting of the introduction of the supernatant concentration phase. The raw HC samples also presented arabinan contents ranging from 9% to 11.5%, confirming the arabinoxylan-type HC structure evidenced by multiple authors [80,81]. Galactose residues present in hemicelluloses’ hydrolysate also pointed to the existing galactan-type branches (2–5%) on the xylan backbone of alkaline CS HC samples [82]. The raw HC samples also yielded glucose upon hydrolysis, but the possible source is questionable since the oligomers that include anhydro-gluco-pyranose units may also precipitate under ethanol addition. The purity of HC samples is also affected by the AIL presence which was a result of co-precipitation of HCs and lignin.

### 3.3. FTIR Spectroscopy Characterization of the Lignin and Hemicellulose Samples

FTIR characterization has been the method of choice to study and investigate either a single component or the structure of different materials, including vegetal biomass, and to monitor the changes that appear in the composition and structure of materials during chemical treatments [83,84]. In this study, the structural features in lignin and hemicellulose samples were analyzed by FTIR spectroscopy. A complete description of the main absorption bands of hemicellulose and lignin is given in Table 6 for lignin samples [84,85,86,87,88,89,90] and Table 7 for hemicelluloses [84,85,90,91].

The FTIR spectra of lignin and hemicellulose samples are presented in Figures S6 and S7, respectively, in the Supplementary Materials.

### 3.4. The Antioxidant Activity of Lignin, HCs, and ERLSs

The potential use of lignin, hemicelluloses, and their various degradation products as antioxidants in different situations has been extensively studied [92,93,94]. Table 8 shows the values obtained for antioxidant activities as IC_50_ values obtained for the purified lignin samples, hemicellulose samples, and ERLS solids. In similar conditions, the DPPH assay for ascorbic acid led to a value of 0.016 mg/mL. Lignin samples showed relatively similar values for IC_50_, with the HWT-treated sample showing a minor increase in comparison to the rest of the samples. Lignin separated from the black liquor was little differentiated from lignin samples separated from alkaline extraction liquors. The determined values strongly differed from those reported by Piccinino et al. for lignin fractions of softwood Kraft lignin (11.1–19.5 ug/mL) [95], but were similar to those mentioned for various organosolv lignin and Kraft lignin samples separated from poplar wood (IC_50_ values ranging from 0.5–3.0 mg/mL), as reported by Lu et al. [96], and to those of Duan et al. (0.4–0.8 mg/mL) for formic acid lignin from *Phragmites australis* [97].

The antioxidant activity of crude HCs was found to be in general about 10 times lower than that of lignin samples, ranging from 1.55 mg/mL for the HCs isolated from the AE SP black liquor to about 3 mg/mL for AE liquor lignin. In general, the obtained IC50 values did not correlate with the AIL content of HC samples. However, similar results in terms of antioxidant activity values were reported by Borovkova for HC samples extracted from spruce [98] and also by Rivas et al. for E. globulus and P. pinaster and rice husks (0.25–5 mg/mL) [92].

The antioxidant activity of the organic solids contained in the HWT liquor showed a value of IC_50_ equal to 0.71. Furthermore, we also tested the antioxidant activity of the solids dissolved in the liquid stream resulting after ethanol recovery, which showed minor antioxidant activity when compared to the rest of the products (lignin and hemicelluloses).

### 3.5. Mass Balance Analysis

**Figure 5:**
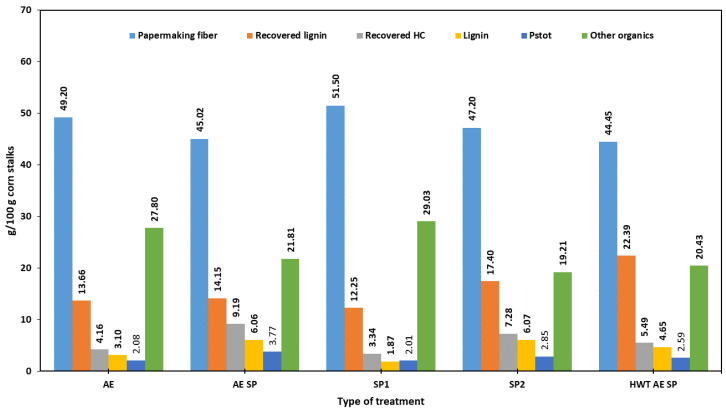
Mass balance for the studied configurations of CS treatment.

Figure 5 displays the amounts of papermaking fibers and raw co-products generated in each experimental set-up. At first sight, and from the perspective of the total pulp yield, the most favorable situation is to simply use either SP1 or AE as the method of separation for CS components. Both of these choices are limited by the high amounts of rejects. The next obvious choice would be SP2, which yielded better in terms of screen yield. The AE SP and HWT AE SP apparently performed the worst in terms of total yield but were better in terms of sorted yields. However, as previously stated, the amounts of rejects of AE and SP1 consisted of partially delignified CSs which could further release fiber upon efficient additional refining. Since such an option leads to a potential increase in energy consumption, it was not considered for the current study.

Regarding recovered raw lignin, the Figure 5 data indicate that the HWT AE SP configuration leads to the best results, while the AE SP set-up would be much more appropriate to recover HCs. To obtain a better opinion regarding the yields of the co-products, the data in Table 7 should be analyzed as it takes into account purity characteristics. The Table 7 data indicate a competition between AE SP and conventional pulping in terms of pure co-products. In terms of the amounts of removed AIL, the sequence HWT AE SP shows that about 90% of lignin is removed, which is the highest removal value. In contrast, SP2 showed the lowest removal rate. Meanwhile, the highest lignin recovery rate, computed as a ratio between the amount of AIL as g/100 g CS and the amount of removed lignin in g/100 g CS, also was shown to be obtained by the HWT AE SP configuration (52%), followed by SP2 with a recovery yield of about 42.

The reported values for the specific amounts of HCs (as pure polysaccharides) seem similar to those reported by other studies which took into consideration the AE of aspen wood [99] (5 g/100 g of wood) or eucalypt wood (6–10 g/100 g wood) [100].

Conventional pulping demonstrated lower values when chemical consumption was taken into consideration. In terms of potential COD release, the data in Table 9 indicated similar values for the studied configurations except in the case of HWT AE SP.

Analyzing earlier studies has shown that, despite variations in treatment procedures or objectives, efforts have produced comparable results. In such a context, it is worth mentioning the results of Liang et al. [64] who investigated the treatment of CSs with preliminary acid hydrolysis and further alkaline sulfite pulping with the aim of co-producing sugars, papermaking fibers, and lignosulfonate-based surfactant agents and reported a maximum yield of sugars of 28.7 g/100 g CS. These sugars had resulted as hydrolysis products and were present as either monomers or oligomers in post-hydrolysis liquors. The reported process conditions were much more severe (140–150 °C and hydrolysis time of up to 3 h) but according to the authors, no severe consequences were noticed for the strength properties.

Serna-Loaiza et al. mention values of 8.9 g/L xylose in a liquid hot water treatment (LHW) of wheat straw that resulted in a stream [36]. That study’s reported results mention a holding time of 180 min and a temperature of 180 °C, which is also very severe in comparison with our study. In a sequel study with similar objectives, Serna-Loaiza et al. [35] mentioned even higher concentrations of hemicellulosic sugars (12–18 g/L)—LHW pretreatment was carried out at 160 °C with a holding time of 90 min. The obtained papermaking fibers were used to produce paper sheets by mixing with birch Kraft pulp and were reported to have considerably lower values than those presented in the current work.

Malik et al. used a combination of treatments such as alkali extraction followed by alkaline pulping in an attempt to valorize wheat straw [101]. That study reported values of 2.5% total reducing sugars content in the alkali extraction liquor, resulting from experiments that involved 60 min process time at a temperature of 100 °C. The quality of the obtained pulps in terms of strength properties was affected by the nature of the treatment. In such a context, the tensile index values ranged from 20 to 32 Nm/g.

When discussing the possibility of lignin removal, Vakkilainen and Välimäki showed that the removal of lignin from the black liquor may be used within certain limits since significant drops in the heating value also occur [102]. Hamaguchi et al. also examined the possible removal of hemicellulose and lignin by preliminary treatments and found that this has a negative impact on the heating value of the resulting black liquor [103]. In our study, we analyzed the possibility of removing both lignin and hemicelluloses, but a study on the effect on the heating value was not possible. However, the advantages of alkaline treatment as a pulping preliminary can help in modifying the silicon mass balance and create opportunities to improve the operation of the chemical recovery.

## 4. Conclusions

Corn stalks were fractionated into papermaking pulp, lignin, and hemicelluloses using various procedures involving sodium hydroxide as the main chemical reagent. The studied CS treatment configurations showed different results in terms of pulp yield and the amounts of recovered lignin and HCs. Soda pulping was the most effective method for maximizing the pulp yield, while alkaline extraction combined with soda pulping produced the best results in terms of lignin and HC recovery, and reasonable pulp yields. The strength tests revealed that the obtained pulp was suitable for packaging papermaking in all the experiments, with the best results obtained with the AE and SP combination. The treatment type affected the purity of the lignin but not its antioxidant activity. Data for mass balance and additional efficiency comparisons were provided by chemical analyses of CSs, treated CSs, pulp, and liquid-generated streams. It was found that each treatment route has both advantages and drawbacks in terms of product quality and yield, as well as for reagent consumption. Depending on the treatment sequence chosen, 3.5 to 6.2 g/100 g CS of relatively pure HCs can be produced, representing 15 to 25% of the original HCs in CSs.

## Figures and Tables

**Table 1:** Different technological approaches for advanced corn agri-waste valorization.

Product	Pretreatment		Extraction		Ref.
	Type	Parameters	Method	Key Factor	
Biogas	acidic	T = 100, 108, 116, 124, and 132 °C t = 5, 17.5, 30, 42.5, and 50 min H_2_SO_4_ conc. = 0, 0.75, 1.5, 2.25, and 3% (*v*∙*v*^−1^) dry mass = 1, 5.5, 10, 14.5, and 19 g	co-digestion	swine manure, mesophilic anaerobic microorganisms	[28]
	alkaline, H_2_O_2_	T = 36, 47, and 58 °C t = 32.5, 60, and 87.5 min H_2_O_2_ conc. = 4, 8, and 12% (*v*∙*v*^−1^) dry mass = 3, 5, and 7 g stirring = 130, 150, and 170 rpm			
Organic acids	acidic	50 g dry mass in 1 L H_2_SO_4_ (1 M) or CH_3_-COOH (1 M), 24 h, room temperature, static	fermentation	microbial consortium	[29]
	alkaline	50 g dry mass in 1 L NaOH (0.4 N) or NH_4_OH (1 M), 24 h, room temperature, static			
	steam explosion	20 g dry mass, 1 L reactor, saturated steam P = 0.65–0.75 MPa, T = 165 °C, t = 3 min			
Ethanol	acidic	H_2_SO_4_ conc. 2% (*w*/*v*) T = 121 °C, t = 60 min dry mass = 10% (*w*/*v*)	enzymatic hydrolysis and fermentation	cellulase, Saccharomyces cerevisiae	[20]
Acetone–butanol–ethanol	alkaline	NaOH conc. = 0.5, 1, 1.5, and 2% (*w*/*v*) T = 80, 100, and 120 °C t = 60 min dry mass = 10% (*w*/*v*)	enzymatic hydrolysis and fermentation	cellulase, Clostridium acetobutylicum	[30]
Cellulose	enzymatic	Xylanase, laccase T = 40 °C, 0.1 mol∙L^−1^ acetic acid/sodium acetate buffer solution, t = 12 h	alkaline treatment	sodium chlorite, acetic acid	[31]
Reducing sugar	alkaline	60 g dry mass, 2.40 g NaOH and 3.60 g CaO dissolved in 60 mL distilled water, steamed at 50 Hz and 2200 W, t = 0.50, 1.00, 1.50, and 2.00 h	enzymatic hydrolysis	cellulase and β-glucanase	[32]
Ethyl levulinate	ionic liquid: [B2-HEA][OAc]	1 g dry mass, 10 g of [B2-HEA][OAc], T = 110–150 °C	catalytic conversion	[C_3_H_6_SO_3_Hmim]HSO_4_	[33]
Succinic acid	organosolv: 70% ethanol	T = 170 °C, t = 1 h s:L = 1:10, ethanol 70%	enzymatic hydrolysis/fermentation	Actinobacillus succinogenes	[34]

T = temperature, P = pressure, t = time, conc. = concentration, s:L = solid/liquid ratio.

**Table 2:** Purity, recovery yield, and viscosity average degree of polymerization of HC samples isolated from BL.

Type of Sample	SF	HC		
		P (%)	RY (%)	DP_v_
AE	2.77	86.45	59.25	247
AE SP (BL)	3.98	89.45	63.83	265
SP1	3.92	88.55	55.69	260
SP2	3.94	84.87	69.81	243
HWT AE SP (BL)	4.01	84.65	60.41	163

P = purity of polysaccharides calculated as the sum of contents of glucan, xylan, galactan, and arabinan; RY = recovery yield calculated as the ratio between the recovered amount of polysaccharides and the amount of removed polysaccharides.

**Table 3:** Mechanical properties of obtained pulps.

Type of Sample	SF	TI (N·m/g)	BI (kPa·m^2^/g)	SCTI (N·m/kg)	CMTI (N·m/g)	DP_v_	Total Pulp Yield (%)	Rejects (%)
AE	2.77	70.4	3.79	44.51	1.33	n.d.	49.2	25.22
AE SP	3.98	77.1	4.72	45.10	1.72	950	45.03	4.52
SP1	3.92	68.2	3.59	39.97	1.74	1080	51.47	22.22
SP2	3.94	72.7	4.61	45.78	1.33	1050	47.2	10.05
HWT AE SP	4.01	74.5	4.46	45.23	1.49	810	44.4	3.45

**Table 4:** Chemical composition of the liquid streams.

Experiment Type	Sample Type	pH	Cond. (mS/cm)	AIL (g/L)	ASL (g/L)	TL (g/L)	Xyl. (g/L)	PS_total_ (g/L)	DMC (g/L)	IMC (g/L)	OMC (g/L)
HWT	HWT	5.5	5.2	0.32	0.61	0.93	0.55	1.01	3.48	1.195	2.28
AE	AE	12.85	33.2	3.73	0.79	4.52	2.25	4.68	27.89	13.24	14.65
AE SP	AE	12.75	32.8	3.56	0.74	4.30	2.24	4.63	27.41	13.42	13.99
	BL	12.61	26.5	6.55	1.07	7.62	4.44	7.92	37.38	13.97	23.41
SP1	BL	10.11	21.3	10.32	1.28	11.60	4.65	10.90	50.66	15.67	34.99
SP2	BL	12.72	29.3	6.95	0.99	7.94	3.60	7.19	34.59	14.29	20.3
HWT AE SP	AE	12.66	32.3	4.88	0.76	5.64	2.86	4.78	26.66	13.57	13.09
	BL	12.40	28.2	9.37	1.17	10.54	5.83	9.83	39.3	17.01	22.29

DMC (g/L) represents the dry matter content, IMC (g/L) and OMC (g/L) stand for the inorganic and organic matter content. TL is the total lignin as a sum of acid-insoluble lignin—AIL and acid-soluble lignin—ASL. HWT designates the liquor obtained after HWT treatment, while AE designates the alkaline extraction liquor, and finally, BL was used to designate the black liquor. Similar notations were used to designate corresponding lignin and hemicellulose samples separated from the above-mentioned liquid streams.

**Table 5:** Chemical composition of ethanol recovery liquid streams.

Experiment Type	Sample Type	ASL (g/L)	Phenols (g/L)	Xyl. (g/L)	PS_total_ (g/L)	DMC (g/L)	IMC (g/L)	OMC (g/L)
AE	AE	5.37	4.06 × 10^−3^	0.82	3.47	49.9	23.39	26.51
AE SP	AE	5.25	3.93 × 10^−3^	0.8	3.65	51.2	23.05	28.15
	BL	6.48	16.38 × 10^−3^	0.82	3.95	45.15	20.26	24.58
SP1	BL	3.74	7.00 × 10^−3^	0.79	4.02	48.27	21.03	27.24
SP2	BL	6.07	16.16 × 10^−3^	0.60	2.85	40.01	18.64	21.37
HWT AE SP	AE	4.90	6.78 × 10^−3^	0.92	2.91	40.94	19.77	21.17
	BL	6.00	35.78 × 10^−3^	0.64	3.23	38.20	17.73	20.47

**Table 6:** Absorption bands of lignin in the FTIR spectra of corn stalks.

Wavenumber (cm^−1^)	Functional Groups
3414	O–H stretching (aliphatic and phenolic structures)
2937	CH_2_ asymmetric vibration (methylene group, guaiacyl-syringyl)
2846–3000	C–H stretch (methoxyl group and methylene (CH_2_) group)
1713	C=O carbonyl stretching (conjugated ketone and carboxylic groups)
1651	Conjugated C–O and absorbed O–H (degradation of hydroxycinnamic moieties in lignin)
1597, 1513, 1423	Aromatic skeletal vibrations
1463	C–H, CH_2_ deformations stretching in lignin and xylan
1328	C–O of syringyl ring
1265	C–O stretching (coniferyl groups or guaiacyl ring breathing with C–O stretching
1219	Sinapyl and p-coumaryl groups
1154	CH stretching (aromatic ring-guaiacylic)
1124	Aromatic C–H in-plane deformation (syringyl type)
837, 915	C–H out of plane (aromatic rings)

**Table 7:** Absorption bands of hemicelluloses in the FTIR spectra of corn stalks.

Wavenumber (cm^−1^)	Functional Groups
3412	O-H stretch, H-bonded (alcohols, phenols)
2878–2924	Symmetric and asymmetric C–H starch, CH_2_ (methylene), and CH_3_ (methyl) groups
1712–1774	C=O stretching bands in acetyl fragments (ketone/aldehyde and free ester)
1634	Bending vibrations of water
1515	Lignin (minimal amounts in the hemicellulosic fractions)
1463, 1422, 1385, 1323	Carbonyl groups in uronic acid or acetyl groups
1250	C=O and C–O of acetyl and uronic ester groups
1163, 1072 and 990	Arabinoxylans and arabinose side chains
1045	C–O and C–C stretching and the glycosidic bond stretching (xylan) and pyranose ring
896	Glycosidic linkages (xylose units)
700–400	Bending vibrations of hydroxyl groups (out of plane)

**Table 8:** The antioxidant activity of purified lignin, hemicelluloses, and ERLS solids.

Experiment Type	Sample Origin	IC_50_ (mg/mL)		
		Lignin	Hemicelluloses	ERLS Solids
AE	AEL	0.36	3.22	13.01
AE SP	AEL	0.35	3.11	14.60
	BL	0.36	1.55	13.69
SP1	BL	0.33	2.19	16.27
SP2	BL	0.37	1.82	12.44
HWT AE SP	BL	0.37	2.21	12.24
	BL	0.39	2.41	11.59

**Table 9:** Amounts of co-products generated in the studied CS processing scenarios and consumption of chemicals.

Experiment Type		PRL (g/100 g)	PR HC (g/100 g)	Chemical Consumption g/100 g CS			COD g/100 g CS
				NaOH	AA	EtOH	
AE	AEL	6.61	3.60	20	57.1	55.4	16.22
AE SP	AEL	1.35	1.73	20	27.3	55.4	
	BL	5.86	3.47		25.7	55.4	
	total AE SP	7.21	5.20		53.0	110.8	15.43
SP1	BL	5.89	2.96	10	12.6	55.4	13.37
SP2	BL	7.62	6.18	20	48.3	55.4	14.78
HWT AE SP	AEL	3.26	1.38	20	29.0	55.4	
	BL	7.23	3.40		23.7	55.4	
	total HWT AE SP	10.49	4.78		52.7	110.8	23.34

PRL = pure recovered lignin expressed as AIL; PR HC = pure recovered hemicelluloses expressed as the sum of contents of glucan, xylan, galactan, and arabinan; AA = acetic acid (pure); EtOH = pure ethanol.

## Data Availability

Data are contained within the article and Supplementary Materials.

## Supplementary Materials

The following supporting information can be downloaded at: https://www.mdpi.com/article/10.3390/polym16111542/s1, Table S1: A glimpse at the options for corn waste valorization; Figures S1–S5: Photo samples of laboratory paper sheets; Figure S6: FTIR spectra of lignin samples; Figure S7: FTIR spectra of hemicelluloses samples.

## References

[1] García-Lara S., Serna-Saldivar S.O., Serna-Saldivar S.O. (2019). Chapter 1—Corn History and Culture. Corn.

[2] Fu Y., Zhang J., Guan T. (2023). High-Value Utilization of Corn Straw: From Waste to Wealth. Sustainability.

[3] Arnold L.K. (1929). Corn-Stalks and Cobs in Industry. Sci. Mon..

[4] Ferraretto L.F., Shaver R.D., Luck B.D. (2018). Silage review: Recent advances and future technologies for whole-plant and fractionated corn silage harvesting. J. Dairy Sci..

[5] Tiammee S., Likasiri C. (2020). Sustainability in corn production management: A multi-objective approach. J. Clean. Prod..

[6] Ojediran J.O., Adeboyejo K., Adewumi A.D., Okonkwo C.E. (2020). Evaluation of briquettes produced from maize cob and stalk. IOP Conf. Ser. Earth Environ. Sci..

[7] Enawgaw H., Tesfaye T., Yilma K.T., Limeneh D.Y. (2023). Multiple Utilization Ways of Corn By-Products for Biomaterial Production with Bio-Refinery Concept; a Review. Mater. Circ. Econ..

[8] Kamusoko R., Jingura R.M., Parawira W., Chikwambi Z. (2021). Strategies for valorization of crop residues into biofuels and other value-added products. Biofuels Bioprod. Biorefining.

[9] Kukreti N., Kag S., Ruhal R., Kataria R., Nandabalan Y.K., Garg V.K., Labhsetwar N.K., Singh A. (2022). A Sustainable Biorefinery Approach to Valorize Corn Waste to Valuable Chemicals. Zero Waste Biorefinery.

[10] Miranda M.T., García-Mateos R., Arranz J.I., Sepúlveda F.J., Romero P., Botet-Jiménez A. (2021). Selective Use of Corn Crop Residues: Energy Viability. Appl. Sci..

[11] Rame R., Purwanto P., Sudarno S. (2023). Biotechnological approaches in utilizing agro-waste for biofuel production: An extensive review on techniques and challenges. Bioresour. Technol. Rep..

[12] Santolini E., Bovo M., Barbaresi A., Torreggiani D., Tassinari P. (2021). Turning Agricultural Wastes into Biomaterials: Assessing the Sustainability of Scenarios of Circular Valorization of Corn Cob in a Life-Cycle Perspective. Appl. Sci..

[13] Sokhansanj S., Turhollow A., Cushman J., Cundiff J. (2002). Engineering aspects of collecting corn stover for bioenergy. Biomass-Bioenergy.

[14] Zabed H.M., Akter S., Yun J., Zhang G., Zhao M., Mofijur M., Awasthi M.K., Kalam M.A., Ragauskas A., Qi X. (2023). Towards the sustainable conversion of corn stover into bioenergy and bioproducts through biochemical route: Technical, economic and strategic perspectives. J. Clean. Prod..

[15] Santana Á.L., Meireles M.A.A. (2023). Valorization of Cereal Byproducts with Supercritical Technology: The Case of Corn. Processes.

[16] Melati R.B., Shimizu F.L., Oliveira G., Pagnocca F.C., de Souza W., Sant’Anna C., Brienzo M. (2019). Key Factors Affecting the Recalcitrance and Conversion Process of Biomass. BioEnergy Res..

[17] Zhao X., Zhang L., Liu D. (2012). Biomass recalcitrance. Part I: The chemical compositions and physical structures affecting the enzymatic hydrolysis of lignocellulose. Biofuels Bioprod. Biorefining.

[18] Zhao X., Zhang L., Liu D. (2012). Biomass recalcitrance. Part II: Fundamentals of different pre-treatments to increase the enzymatic digestibility of lignocellulose. Biofuels Bioprod. Biorefining.

[19] Zhang YaNing Z.Y., Ghaly A., Li BingXi L.B. (2012). Physical properties of corn residues. Am. J. Biochem. Biotechnol..

[20] Li P., Cai D., Luo Z., Qin P., Chen C., Wang Y., Zhang C., Wang Z., Tan T. (2016). Effect of acid pretreatment on different parts of corn stalk for second generation ethanol production. Bioresour. Technol..

[21] Li Z., Zhai H., Zhang Y., Yu L. (2012). Cell morphology and chemical characteristics of corn stover fractions. Ind. Crop. Prod..

[22] Wang Y., Xu X., Xue H., Zhang D., Li G. (2021). Physical–chemical properties of cell wall interface significantly correlated to the complex recalcitrance of corn straw. Biotechnol. Biofuels.

[23] Berchem T., Roiseux O., Vanderghem C., Boisdenghien A., Foucart G., Richel A. (2017). Corn stover as feedstock for the production of ethanol: Chemical composition of different anatomical fractions and varieties. Biofuels Bioprod. Biorefining.

[24] Zhu Y., Lee Y.Y., Elander R.T. (2005). Optimization of dilute-acid pretreatment of corn stover using a high-solids percolation reactor. Appl. Biochem. Biotechnol..

[25] Puițel A.C., Suditu G.D., Danu M., Ailiesei G.-L., Nechita M.T. (2022). An Experimental Study on the Hot Alkali Extraction of Xylan-Based Hemicelluloses from Wheat Straw and Corn Stalks and Optimization Methods. Polymers.

[26] Kang K., Nanda S., Sun G., Qiu L., Gu Y., Zhang T., Zhu M., Sun R. (2019). Microwave-assisted hydrothermal carbonization of corn stalk for solid biofuel production: Optimization of process parameters and characterization of hydrochar. Energy.

[27] Zhang H., Wu J. (2023). Statistical Optimization of Tween-80-Assisted Potassium Hydroxide Pretreatment and Enzymatic Hydrolysis for Enhancing Sugar Yields from Corn Cob. Fermentation.

[28] Venturin B., Frumi Camargo A., Scapini T., Mulinari J., Bonatto C., Bazoti S., Pereira Siqueira D., Maria Colla L., Alves S.L., Paulo Bender J. (2018). Effect of pretreatments on corn stalk chemical properties for biogas production purposes. Bioresour. Technol..

[29] Guo P., Mochidzuki K., Cheng W., Zhou M., Gao H., Zheng D., Wang X., Cui Z. (2011). Effects of different pretreatment strategies on corn stalk acidogenic fermentation using a microbial consortium. Bioresour. Technol..

[30] Cai D., Li P., Luo Z., Qin P., Chen C., Wang Y., Wang Z., Tan T. (2016). Effect of dilute alkaline pretreatment on the conversion of different parts of corn stalk to fermentable sugars and its application in acetone–butanol–ethanol fermentation. Bioresour. Technol..

[31] Lou C., Zhou Y., Yan A., Liu Y. (2022). Extraction cellulose from corn-stalk taking advantage of pretreatment technology with immobilized enzyme. RSC Adv..

[32] Liu C., Liu M., Wang P., Chang J., Yin Q., Zhu Q., Lu F. (2020). Effect of steam-assisted alkaline pretreatment plus enzymolysis on converting corn stalk into reducing sugar. Renew. Energy.

[33] Wang Y., Zheng X., Lin X., Liu X., Han D., Zhang Q. (2024). Total component transformation of corn stalk to ethyl levulinate assisted by ionic liquid pretreatment. Cellulose.

[34] Buyukoztekin G.K., Buyukkileci A.O. (2024). Enzymatic hydrolysis of organosolv-pretreated corncob and succinic acid production by Actinobacillus succinogenes. Ind. Crop. Prod..

[35] Serna-Loaiza S., Adamcyk J., Beisl S., Miltner M., Friedl A. (2022). Sequential Pretreatment of Wheat Straw: Liquid Hot Water Followed by Organosolv for the Production of Hemicellulosic Sugars, Lignin, and a Cellulose-Enriched Pulp. Waste Biomass Valoriz..

[36] Serna-Loaiza S., Zikeli F., Adamcyk J., Friedl A. (2021). Towards a wheat straw biorefinery: Combination of Organosolv and Liquid Hot Water for the improved production of sugars from hemicellulose and lignin hydrolysis. Bioresour. Technol. Rep..

[37] Puițel A.C., Balan C.D., Ailiesei G.-L., Drăgoi E.N., Nechita M.T. (2023). Integrated Hemicellulose Extraction and Papermaking Fiber Production from Agro-Waste Biomass. Polymers.

[38] (2004). Pulps—Preparation of Laboratory Sheets for Physical Testing—Part 2: Rapid-Köthen Method.

[39] (1979). Pulps—Laboratory Beating—Part 3: Jokro Mill Method.

[40] (2008). Paper and board—Determination of Tensile Properties—Part 2: Constant rate of Elongation Method (20 mm/min).

[41] (2014). Paper—Determination of Bursting Strength.

[42] (2008). Paper and Board Compressive Strength. Short-Span Test.

[43] (2018). Corrugating Medium. Determination of the Flat Crush Resistance after Laboratory Fluting. Part 1: A-Flute.

[44] Ziegler-Devin I., Chrusciel L., Brosse N. (2021). Steam Explosion Pretreatment of Lignocellulosic Biomass: A Mini-Review of Theorical and Experimental Approaches. Front. Chem..

[45] Overend R.P., Chornet E. (1987). Fractionation of lignocellulosics by steam-aqueous pretreatments. Philos. Trans. R. Soc. Lond. Ser. A Math. Phys. Sci..

[46] Technical Association of the Pulp and Paper Industry (1988). Water Solubility of Wood and Pulp.

[47] Technical Association of the Pulp and Paper Industry (2007). 204 cm-97, Solvent Extractives of Wood and Pulp.

[48] Technical Association of the Pulp and Paper Industry (2000). Acetone Extractives of Wood and Pulp.

[49] Sluiter A., Hames B., Hyman D., Payne C., Ruiz R., Scarlata C., Sluiter J., Templeton D., Wolfe J. (2008). Determination of total solids in biomass and total dissolved solids in liquid process samples. NREL.

[50] Sluiter A., Hames B., Ruiz R., Scarlata C., Sluiter J., Templeton D., Crocker D. (2008). Determination of structural carbohydrates and lignin in biomass. Lab. Anal. Proced..

[51] Atİk C., Ates S. (2012). Mass balance of silica in straw from the perspective of silica reduction in straw pulp. BioResources.

[52] Okorie N.N., Momoh I.M., Adeeyinwo C.E. (2015). Molybdenum blue method determination of silicon in amorphous silica. Acta Tech. Corviniensis-Bull. Eng..

[53] Farhat W., Venditti R., Quick A., Taha M., Mignard N., Becquart F., Ayoub A. (2017). Hemicellulose extraction and characterization for applications in paper coatings and adhesives. Ind. Crop. Prod..

[54] Koshijima T., Timell T.E., Zinbo M. (1965). The number-average molecular weight of native hardwood xylans. J. Polym. Sci. Part C Polym. Symp..

[55] Salam A., Pawlak J.J., Venditti R.A., El-tahlawy K. (2011). Incorporation of carboxyl groups into xylan for improved absorbency. Cellulose.

[56] (1981). Cellulose in Dilute Solutions—Determination of Limiting Viscosity Number—Part 1: Method in Cupri-Ethylene-Diamine (CED) Solution.

[57] Sluiter A., Hames B., Ruiz R., Scarlata C., Sluiter J., Templeton D. (2006). Determination of sugars, byproducts, and degradation products in liquid fraction process samples. Gold. Nat. Renew. Energy Lab..

[58] (1990). Water quality—Determination of phenol index—4-Aminoantipyrine spectrometric methods after distillation.

[59] (2002). Water quality—Determination of the chemical oxygen demand index (ST-COD) — Small-scale sealed-tube method.

[60] Amarowicz R., Pegg R.B., Ferreira I.C.F.R., Barros L. (2019). Chapter One—Natural Antioxidants of Plant Origin. Advances in Food and Nutrition Research.

[61] Bondet V., Brand-Williams W., Berset C. (1997). Kinetics and Mechanisms of Antioxidant Activity using the DPPH. Free Radical Method. LWT-Food Sci. Technol..

[62] Dai Y., Sun Q., Wang W., Lu L., Liu M., Li J., Yang S., Sun Y., Zhang K., Xu J. (2018). Utilizations of agricultural waste as adsorbent for the removal of contaminants: A review. Chemosphere.

[63] Ding K., Lin H., Liu L., Jia X., Zhang H., Tan Y., Liang X., He Y., Liu D., Han L. (2023). Effect of ball milling on enzymatic sugar production from fractionated corn stover. Ind. Crop. Prod..

[64] Liang J., Li Z., Dai S., Tian G., Wang Z. (2023). Production of hemicelluloses sugars, cellulose pulp, and lignosulfonate surfactant using corn stalk by prehydrolysis and alkaline sulfite cooking. Ind. Crop. Prod..

[65] Pordesimo L.O., Hames B.R., Sokhansanj S., Edens W.C. (2005). Variation in corn stover composition and energy content with crop maturity. Biomass Bioenergy.

[66] Tripathi S.K., Bhardwaj N.K., Ainun Z.M.A., Sapuan S.M., Ilyas R.A. (2023). Chapter 5—Pulping and Papermaking of Cornstalk. Pulping and Papermaking of Nonwood Plant Fibers.

[67] Kim T.H., Lee Y.Y. (2006). Fractionation of corn stover by hot-water and aqueous ammonia treatment. Bioresour. Technol..

[68] Kang X., Wang Y.-Y., Wang S., Song X. (2021). Xylan and xylose decomposition during hot water pre-extraction: A pH-regulated hydrolysis. Carbohydr. Polym..

[69] Xu Y., Sun H., Li X., Zhang D., Yong T. (2015). Method of black liquor combustion to remove silicon from wheat straw pulping. BioResources.

[70] Małachowska E., Dubowik M., Boruszewski P., Łojewska J., Przybysz P. (2020). Influence of lignin content in cellulose pulp on paper durability. Sci. Rep..

[71] Hubbe M.A. (2014). Prospects for maintaining strength of paper and paperboard products while using less forest resources: A review. BioResources.

[72] Hagel S., Schütt F. (2024). Reinforcement Fiber Production from Wheat Straw for Wastepaper-Based Packaging Using Steam Refining with Sodium Carbonate. Clean Technol..

[73] Krogell J., Eränen K., Granholm K., Pranovich A., Willför S. (2014). High-temperature pH measuring during hot-water extraction of hemicelluloses from wood. Ind. Crop. Prod..

[74] Brown M.T., Hart P.W. (2017). Understanding conductivity and soda loss. TAPPI J..

[75] Sasmal S., Mohanty K., Kumar S., Sani R.K. (2018). Pretreatment of Lignocellulosic Biomass Toward Biofuel Production. Biorefining of Biomass to Biofuels: Opportunities and Perception.

[76] Manorma S., André S., Patrícia A., Licínio M.G.-F. (2022). Efficient Recovery of Lignin and Hemicelluloses from Kraft Black Liquor. KnE Mater. Sci..

[77] Andeme Ela R.C., Spahn L., Safaie N., Ferrier R.C., Ong R.G. (2020). Understanding the Effect of Precipitation Process Variables on Hardwood Lignin Characteristics and Recovery from Black Liquor. ACS Sustain. Chem. Eng..

[78] Jose S., Mishra L., Basu G., Kumar Samanta A. (2017). Study on Reuse of Coconut Fiber Chemical Retting Bath. Part II—Recovery and Characterization of Lignin. J. Nat. Fibers.

[79] Hubbe M.A., Alén R., Paleologou M., Kannangara M., Kihlman J. (2019). Lignin recovery from spent alkaline pulping liquors using acidification, membrane separation, and related processing steps: A review. BioResources.

[80] Li R., Yang G., Chen J., He M. (2017). The characterization of hemicellulose extract from corn stalk with stepwise alkali extraction. J. Korea Tech. Assoc. Pulp Pap. Ind..

[81] Weng V., Cardeira M., Bento-Silva A., Serra A.T., Brazinha C., Bronze M.R. (2023). Arabinoxylan from Corn Fiber Obtained through Alkaline Extraction and Membrane Purification: Relating Bioactivities with the Phenolic Compounds. Molecules.

[82] Slama H.B., Chenari Bouket A., Pourhassan Z., Alenezi F.N., Silini A., Cherif-Silini H., Oszako T., Luptakova L., Golińska P., Belbahri L. (2021). Diversity of Synthetic Dyes from Textile Industries, Discharge Impacts and Treatment Methods. Appl. Sci..

[83] Lazar L., Bandrabur B., Tataru-Fărmuş R.-E., Drobotă M., Bulgariu L., Gutt G. (2014). FTIR analysis of ion exchange resins with application in permanent hard water softening. Environ. Eng. Manag. J..

[84] Xu F., Yu J., Tesso T., Dowell F., Wang D. (2013). Qualitative and quantitative analysis of lignocellulosic biomass using infrared techniques: A mini-review. Appl. Energy.

[85] Zhang A., Liu C., Sun R. (2010). Fractional isolation and characterization of lignin and hemicelluloses from Triploid of Populus tomentosa Carr. Ind. Crop. Prod..

[86] Sun X.-F., Jing Z., Fowler P., Wu Y., Rajaratnam M. (2011). Structural characterization and isolation of lignin and hemicelluloses from barley straw. Ind. Crop. Prod..

[87] Gbenebor O.P., Olanrewaju O.A., Usman M.A., Adeosun S.O. (2023). Lignin from Brewers’ Spent Grain: Structural and Thermal Evaluations. Polymers.

[88] Prozil S.O., Evtuguin D.V., Silva A.M.S., Lopes L.P.C. (2014). Structural Characterization of Lignin from Grape Stalks (*Vitis vinifera* L.). J. Agric. Food Chem..

[89] Reyes-Rivera J., Terrazas T., Agusti J. (2024). Lignin Analysis by HPLC and FTIR: Spectra Deconvolution and S/G Ratio Determination. Xylem: Methods and Protocols.

[90] Liu Q., Zhu S. (2023). Fractionation of depectinated sugar beet pulp into cellulose, hemicellulose, and lignin with NaOH/urea/H_2_O and ionic liquid. Int. J. Biol. Macromol..

[91] Kostryukov S.G., Matyakubov H.B., Masterova Y.Y., Kozlov A.S., Pryanichnikova M.K., Pynenkov A.A., Khluchina N.A. (2023). Determination of Lignin, Cellulose, and Hemicellulose in Plant Materials by FTIR Spectroscopy. J. Anal. Chem..

[92] Rivas S., Conde E., Moure A., Domínguez H., Parajó J.C. (2013). Characterization, refining and antioxidant activity of saccharides derived from hemicelluloses of wood and rice husks. Food Chem..

[93] Hassan A.A., Hasanin M.S., Ismail S.A. (2023). Enzymatic valorization of cellulosic and hemicellulosic-based biomasses via the production of antioxidant water-soluble hydrolyzate of maize stalks and the green bio-deinking of mixed office waste paper. Biomass Convers. Biorefinery.

[94] Cassoni A.C., Mota I., Costa P., Vasconcelos M.W., Pintado M. (2022). Effect of alkaline and deep eutectic solvents pretreatments on the recovery of lignin with antioxidant activity from grape stalks. Int. J. Biol. Macromol..

[95] Piccinino D., Capecchi E., Trifero V., Tomaino E., Marconi C., Del Giudice A., Galantini L., Poponi S., Ruggieri A., Saladino R. (2022). Lignin Nanoparticles as Sustainable Photoprotective Carriers for Sunscreen Filters. ACS Omega.

[96] Lu Q., Liu W., Yang L., Zu Y., Zu B., Zhu M., Zhang Y., Zhang X., Zhang R., Sun Z. (2012). Investigation of the effects of different organosolv pulping methods on antioxidant capacity and extraction efficiency of lignin. Food Chem..

[97] Duan X., Wang X., Chen J., Liu G., Liu Y. (2022). Structural properties and antioxidation activities of lignins isolated from sequential two-step formosolv fractionation. RSC Adv..

[98] Borovkova V.S., Malyar Y.N., Sudakova I.G., Chudina A.I., Skripnikov A.M., Fetisova O.Y., Kazachenko A.S., Miroshnikova A.V., Zimonin D.V., Ionin V.A. (2022). Molecular Characteristics and Antioxidant Activity of Spruce (*Picea abies*) Hemicelluloses Isolated by Catalytic Oxidative Delignification. Molecules.

[99] Al-Dajani W.W., Tschirner U. (2007). Alkaline extraction of hemicelluloses from aspen chips and its impact on subsequent kraft pulping. Engineering, Pulping and Environmental Conference 2007.

[100] Vena P.F., García-Aparicio M.P., Brienzo M., Görgens J.F., Rypstra T. (2013). Effect of Alkaline Hemicellulose Extraction on Kraft Pulp Fibers from Eucalyptus Grandis. J. Wood Chem. Technol..

[101] Malik S., Rana V., Joshi G., Gupta P.K., Sharma A. (2020). Valorization of Wheat Straw for the Paper Industry: Pre-extraction of Reducing Sugars and Its Effect on Pulping and Papermaking Properties. ACS Omega.

[102] Vakkilainen E., Välimäki E. (2009). Effect of lignin separation to black liquor and recovery boiler operation. TAPPI Engineering, Pulping & Environmental Conference.

[103] Hamaguchi M., Kautto J., Vakkilainen E. (2013). Effects of hemicellulose extraction on the kraft pulp mill operation and energy use: Review and case study with lignin removal. Chem. Eng. Res. Des..

