# Supplementary material for: The Potential Valorization of Corn Stalks by Alkaline Sequential Fractionation to Obtain Papermaking Fibers, Hemicelluloses, and Lignin—A Comprehensive Mass Balance Approach
Source: Polymers (Basel). 2024 May 30;16(11):1542. doi: 10.3390/polym16111542 (PMC11174482; doi:10.3390/polym16111542)
Supplement: Supplementary file 1 [file polymers-16-01542-s001.zip › polymers-3008072-supplementary.pdf]

Suplimentary material

The potential valorization of corn stalks by alkaline sequential fractionation to papermaking fibers, hemicelluloses, and lignin – a comprehensive mass balance approach

Adrian Cătălin Puițel, Georgiana Bălușescu, Catalin Dumitrel Balan, Mircea Teodor Nechita*

"Gheorghe Asachi" Technical University of Iasi, Faculty of Chemical Engineering and Environmental Protection "Cristofor Simionescu", Bd. Prof. Dimitrie Mangeron, No. 73, 700050, Iaşi, România; e-mail: adrian-catalin.puitel@academic.tuiasi.ro (ACP); georgiana.balusescu@gmail.com (GB); catalin-dumitrel.balan@academic.tuiasi.ro (CDB), mircea-teodor.nechita@academic.tuiasi.ro (MTN)

* Correspondence: mircea-teodor.nechita@academic.tuiasi.ro

Table S1. A glimpse on the options for corn waste valorisation

		**Products**	**Plant component**	**Processing degree**	**Ref.**
**Traditional approach**		silage	stalks, leaves, husks	*low* (mechanical, biochemical)	[1]
		fertilizer	whole plant	low (mechanical)	[2]
		fuel	stalks, cobs	*low* (mechanical)	[3]
**Modern approach**	Single product	Textile fibers	husks	*high* (biochemical, mechanical)	[4, 5]
		Various composites	husks	*high* (mechanical, chemical)	[6]
		Various chemicals	whole plant	*high* (mechanical, chemical, biochemical)	[7]
		Fertilizers	cobs, husks	*moderate (mechanical, biochemical)*	[8]
		Mushrooms substrate	cobs/stalks	*moderate (mechanical, biochemical)*	[9, 10]
		Adsorbents	cobs/stalks	*moderate (mechanical, thermo-chemical)*	[11]
		Pellets	corn stalk rinds	*moderate (mechanical)*	[12]
	Multiple products	Bio-oil and bio-char	stalks	*high* (mechanical, chemical)	[13]
		Fermentable sugars and lignin	whole plant	*high* (chemical, biochemical)	[14]
		Hemicellulose and paper	stalks	high (mechanical, chemical)	[15]

		Biodegradable film, ethanol, and soda pulp	stalks	*high* (chemical, biochemical)	[16]

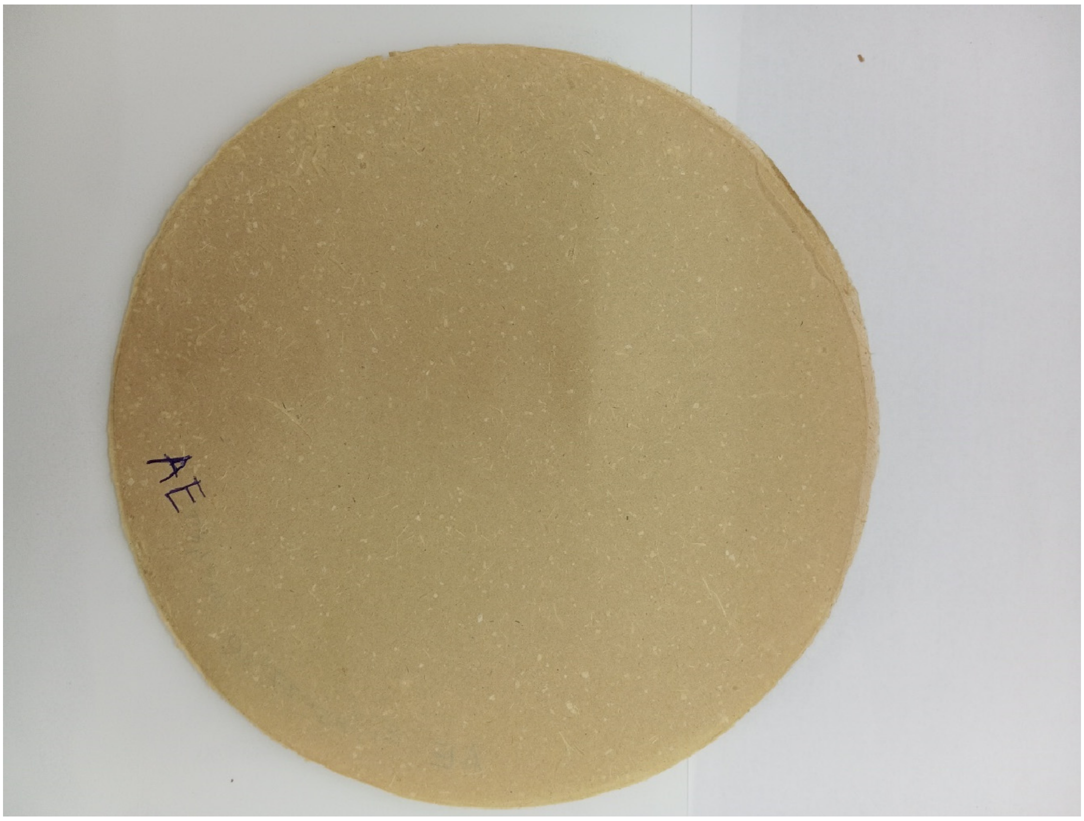

Figure S1 - AE paper sample sheet (*pulp refined up to 45-48 °SR*)

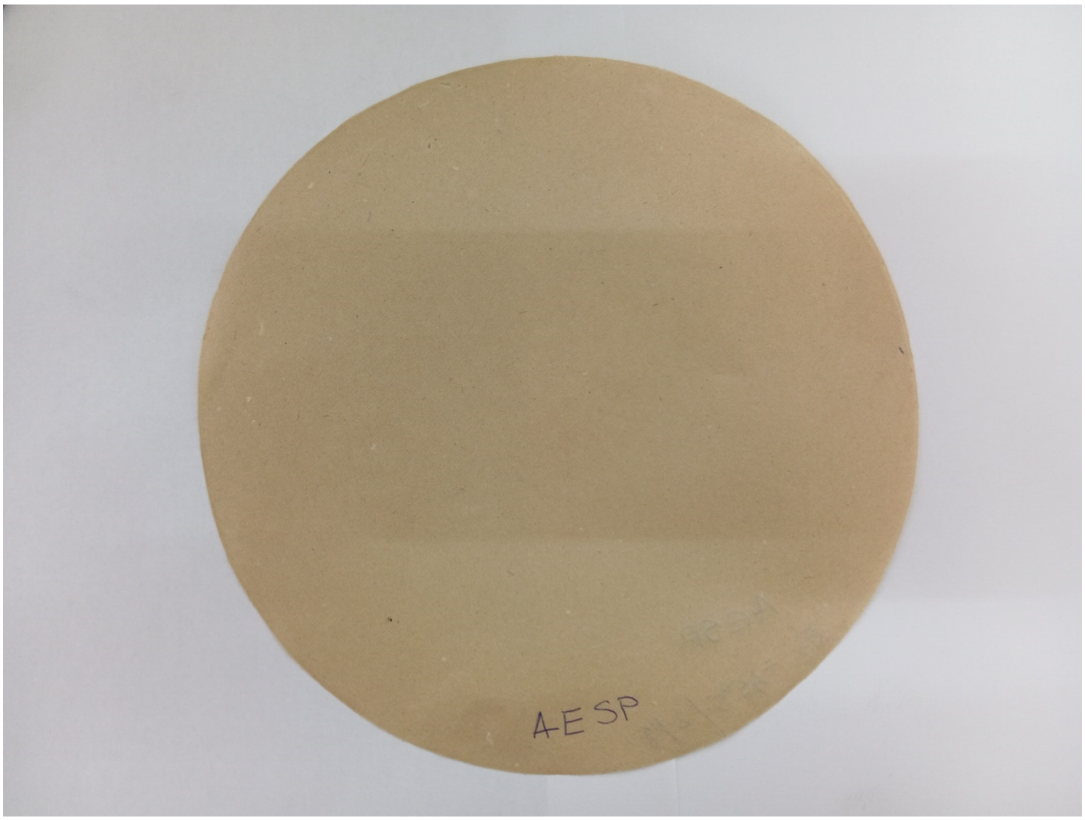

Figure S2 - AE SP paper sample sheet (*pulp refined up to 45-48 °SR*)

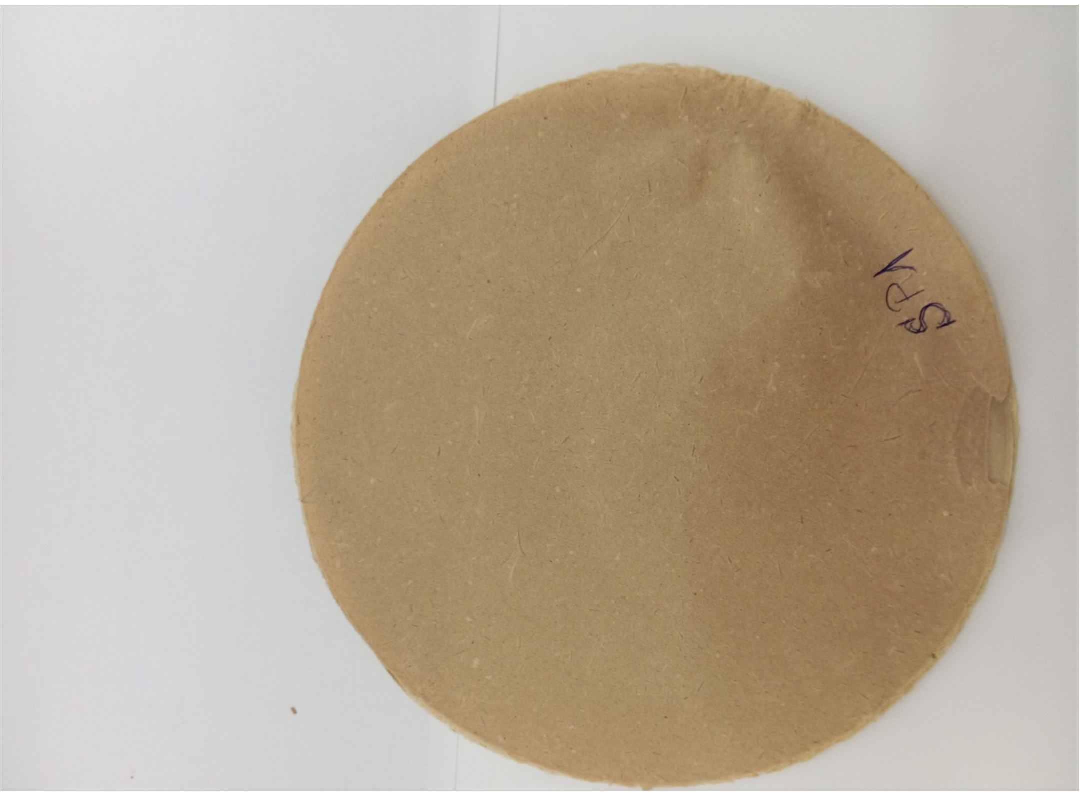

Figure S3 - SP1 paper sample sheet (*pulp refined up to 45-48 °SR*)

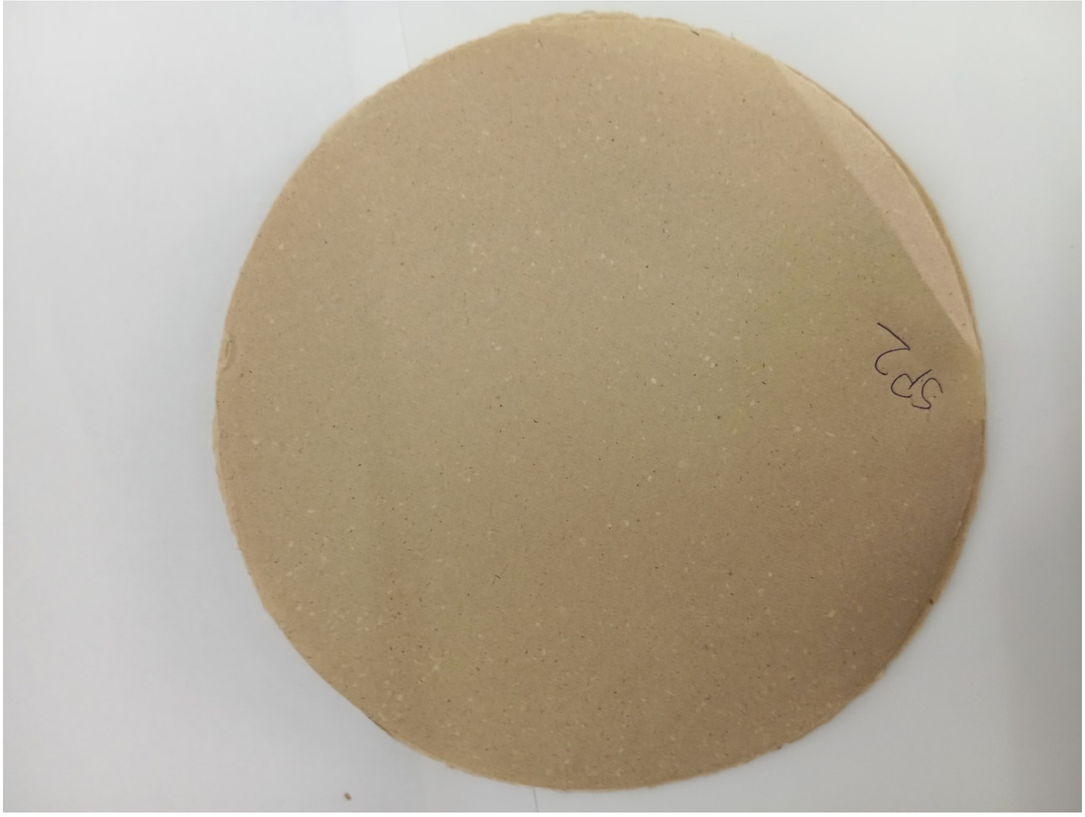

Figure S4 - SP2 paper sample sheet (*pulp refined up to 45-48 °SR*)

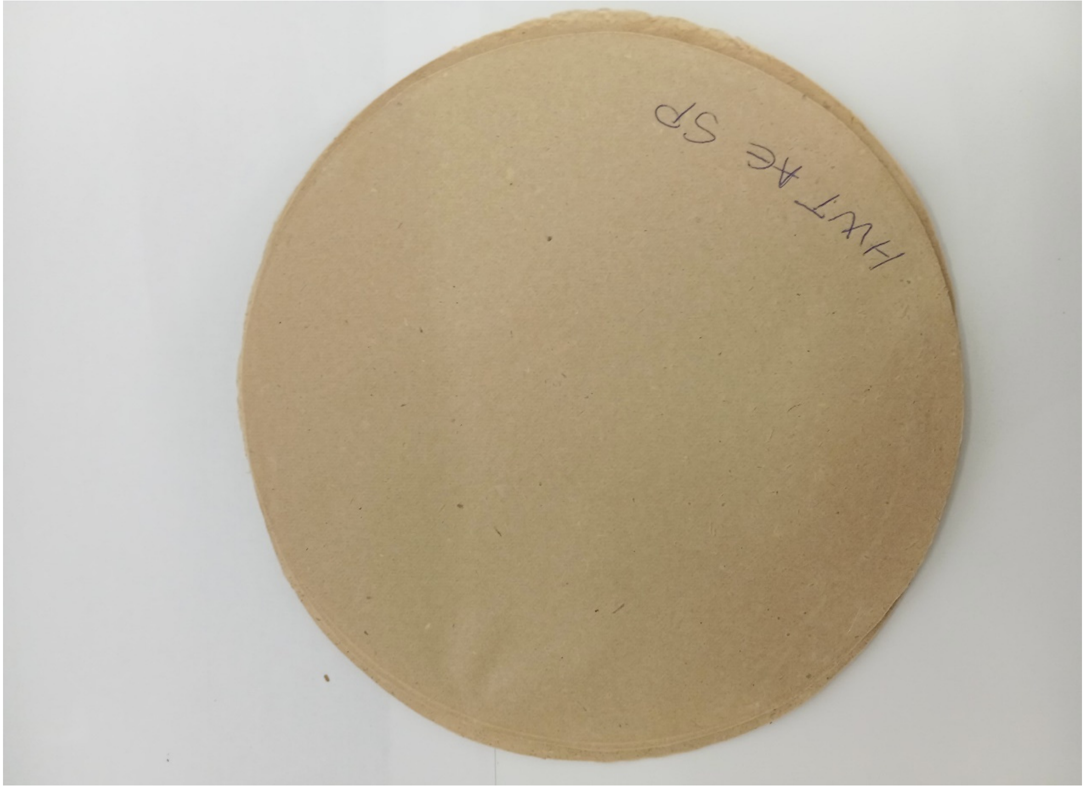

Figure S5 - HWT AE SP paper sample sheet (*pulp refined up to 45-48 °SR*)

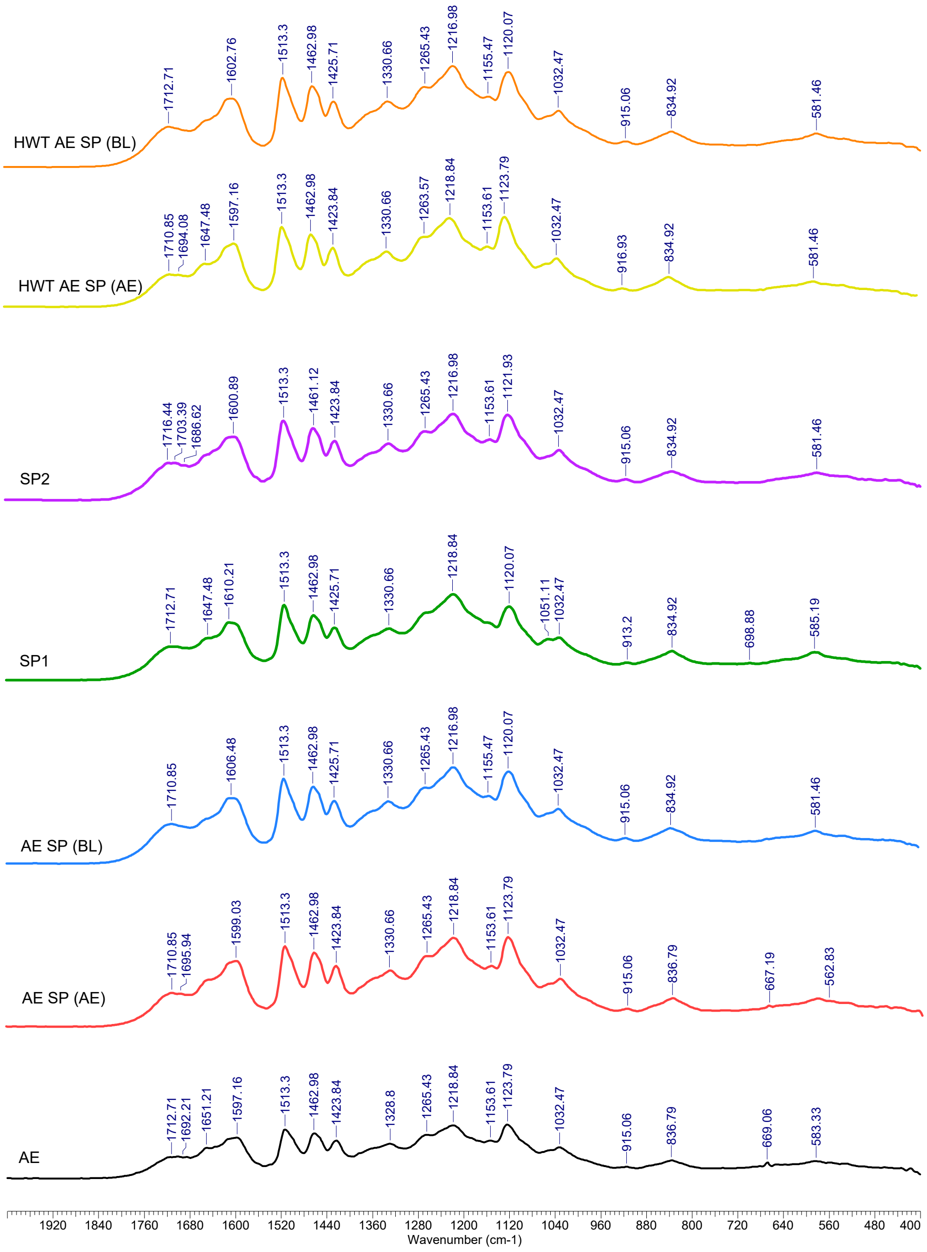

Figure S6 FTIR spectra of lignin samples

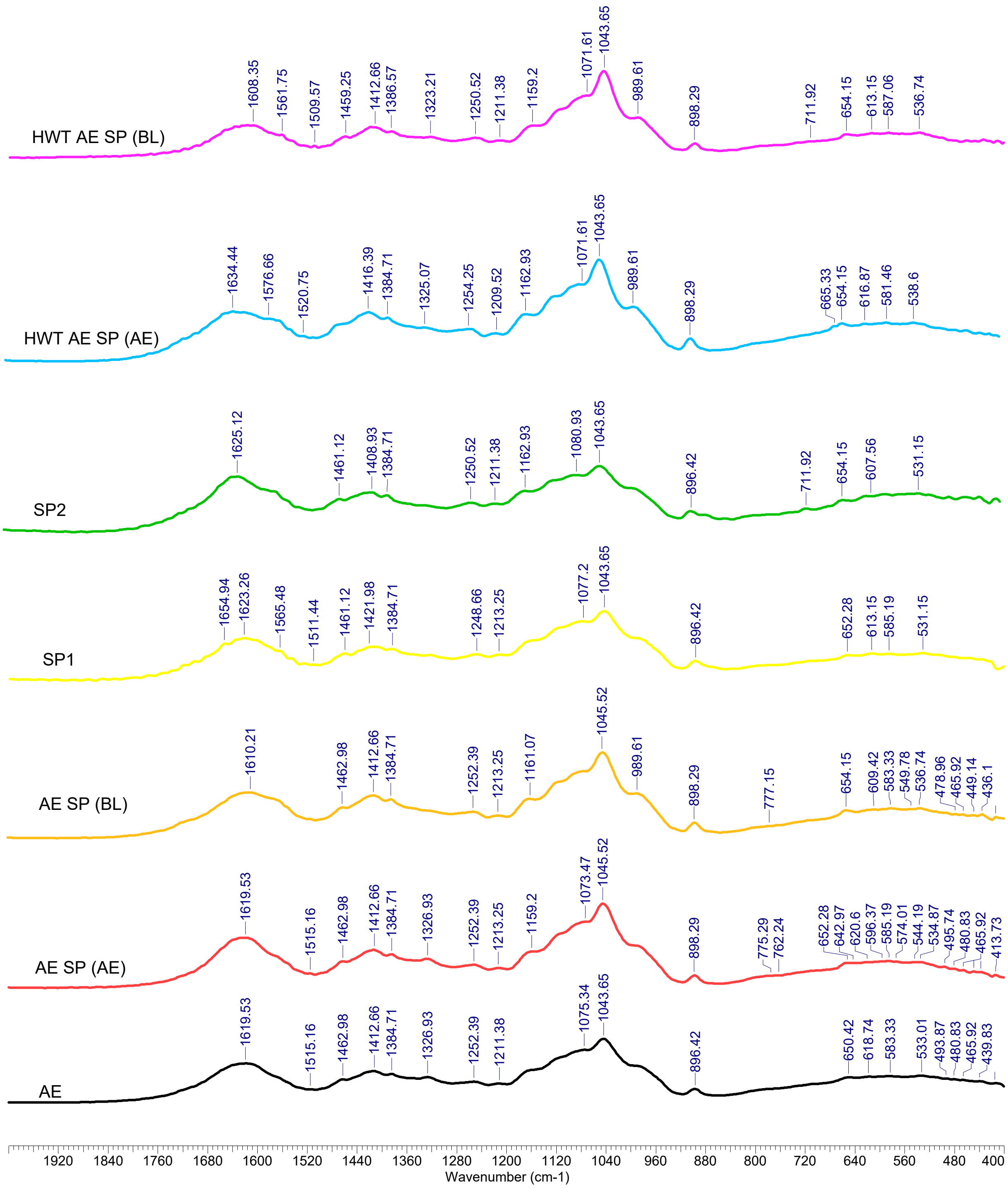

Figure S7 - FTIR spectra of hemicelluloses samples

References

1. Ferraretto, L.F., R.D. Shaver, and B.D. Luck, *Silage review: Recent advances and future technologies for whole-plant and fractionated corn silage harvesting.* Journal of Dairy Science, 2018. **101**(5): p. 3937-3951.
2. Ramm, S., et al., *Comparative Analysis of Mechanical In-Field Corn Residue Shredding Methods: Evaluating Particle Size Distribution and Rating of Structural Integrity of Corn Stalk Segments.* Agriculture, 2024. **14**(2): p. 263.
3. Ojediran, J.O., et al., *Evaluation of briquettes produced from maize cob and stalk.* IOP Conference Series: Earth and Environmental Science, 2020. **445**(1): p. 012052.
4. Patil, H. and A. Athalye, *Valorization of Corn Husk Waste for Textile Applications.* Journal of Natural Fibers, 2023. **20**(1): p. 2156017.
5. Reddy, N. and Y. Yang, *Structure and properties of high quality natural cellulose fibers from cornstalks.* Polymer, 2005. **46**(15): p. 5494-5500.
6. Ratna, A.S., A. Ghosh, and S. Mukhopadhyay, *Advances and prospects of corn husk as a sustainable material in composites and other technical applications.* Journal of Cleaner Production, 2022. **371**: p. 133563.
7. Fu, Y., J. Zhang, and T. Guan, *High-Value Utilization of Corn Straw: From Waste to Wealth.* Sustainability, 2023. **15**(19): p. 14618.
8. Castillo-González, E., et al., *Vermicomposting: A Valorization Alternative for Corn Cob Waste.* Applied Sciences, 2021. **11**(12): p. 5692.
9. Castorina, G., et al., *Characterization and nutritional valorization of agricultural waste corncobs from Italian maize landraces through the growth of medicinal mushrooms.* Scientific Reports, 2023. **13**(1): p. 21148.
10. Zhou, Y., et al., *Evaluation of Corn Stalk as a Substrate to Cultivate King Oyster Mushroom (Pleurotus eryngii).* Horticulturae, 2023. **9**(3): p. 319.
11. Iwuozor, K.O., et al., *A comprehensive review on the sequestration of dyes from aqueous media using maize-/corn-based adsorbents.* Water Practice and Technology, 2023. **18**(12): p. 3065-3108.
12. Liu, D., et al., *Optimization of Process Parameters for Pellet Production from Corn Stalk Rinds Using Box–Behnken Design.* Energies, 2023. **16**(12): p. 4796.
13. Nizamuddin, S., et al., *Solvothermal Liquefaction of Corn Stalk: Physico-Chemical Properties of Bio-oil and Biochar.* Waste and Biomass Valorization, 2019. **10**(7): p. 1957-1968.
14. Lyu, Q., et al., *A multi-product strategy for the fractionation of corn stover based on peracetic acid and maleic acid processing.* Journal of Environmental Chemical Engineering, 2022. **10**(6): p. 108764.
15. Puițel, A.C., et al., *Integrated Hemicellulose Extraction and Papermaking Fiber Production from Agro-Waste Biomass.* Polymers, 2023. **15**(23): p. 4597.
16. Özyürek, Ö. and Y. Çöpür, *Integrated biorefinery for production of biodegradable film, bioethanol, and soda pulp from corn stalks.* BioResources, 2023. **18**(2): p. 2639.
